# Supplementary material for: Bioturbation in the hadal zone
Source: Nat Commun. 2025 Feb 18;16:1401. doi: 10.1038/s41467-025-56627-x (PMC11836284; doi:10.1038/s41467-025-56627-x)
Supplement: Supplementary file 3 — Description of Additional Supplementary Files [file 41467_2025_56627_MOESM3_ESM.pdf]

Description of Additional Supplementary Files

Incipient trace fossil videos

The following videos are provided as separate files:

Supplementary Movie 1. Incipient trace fossil video: *Rhizocorallium*
LINK: WR2 Rhizocorallium.mpg

Supplementary Movie 2. Incipient trace fossil video: *Gyrolithes*
LINK: WR7 Gyrolithes.mpg

Supplementary Movie 3. Incipient trace fossil video: *Zoophycos*-like
LINK: WR8 Zoophycos-like.mpg

Supplementary Movie 4. Incipient trace fossil video: *Pilichnus*
LINK: WR35 Pilichnus.mpg
