# Supplementary material for: Bioturbation in the hadal zone
Source: Nat Commun. 2025 Feb 18;16:1401. doi: 10.1038/s41467-025-56627-x (PMC11836284; doi:10.1038/s41467-025-56627-x)
Supplement: Supplementary file 8 — Reporting Summary [file 41467_2025_56627_MOESM8_ESM.pdf]

nature portfolio

Corresponding author(s): Jussi Hovikoski

Last updated by author(s): Jan 23, 2025

Reporting Summary

Nature Portfolio wishes to improve the reproducibility of the work that we publish. This form provides structure for consistency and transparency in reporting. For further information on Nature Portfolio policies, see our Editorial Policies and the Editorial Policy Checklist.

Statistics

For all statistical analyses, confirm that the following items are present in the figure legend, table legend, main text, or Methods section.

n/a	Confirmed	
☐	☒	The exact sample size (*n*) for each experimental group/condition, given as a discrete number and unit of measurement
☐	☒	A statement on whether measurements were taken from distinct samples or whether the same sample was measured repeatedly
☐	☒	The statistical test(s) used AND whether they are one- or two-sided<br>*Only common tests should be described solely by name; describe more complex techniques in the Methods section.*
☐	☒	A description of all covariates tested
☐	☒	A description of any assumptions or corrections, such as tests of normality and adjustment for multiple comparisons
☐	☒	A full description of the statistical parameters including central tendency (e.g. means) or other basic estimates (e.g. regression coefficient) AND variation (e.g. standard deviation) or associated estimates of uncertainty (e.g. confidence intervals)
☐	☒	For null hypothesis testing, the test statistic (e.g. *F*, *t*, *r*) with confidence intervals, effect sizes, degrees of freedom and *P* value noted<br>*Give P values as exact values whenever suitable.*
☒	☐	For Bayesian analysis, information on the choice of priors and Markov chain Monte Carlo settings
☒	☐	For hierarchical and complex designs, identification of the appropriate level for tests and full reporting of outcomes
☒	☐	Estimates of effect sizes (e.g. Cohen's *d*, Pearson's *r*), indicating how they were calculated

Our web collection on statistics for biologists contains articles on many of the points above.

Software and code

Policy information about availability of computer code

Data collection	-
Data analysis	-

For manuscripts utilizing custom algorithms or software that are central to the research but not yet described in published literature, software must be made available to editors and reviewers. We strongly encourage code deposition in a community repository (e.g. GitHub). See the Nature Portfolio guidelines for submitting code & software for further information.

Data

Policy information about availability of data

All manuscripts must include a data availability statement. This statement should provide the following information, where applicable:

- Accession codes, unique identifiers, or web links for publicly available datasets
- A description of any restrictions on data availability
- For clinical datasets or third party data, please ensure that the statement adheres to our policy

The main data set is presented in the main article and as supplementary items. The supporting C, N and grain size data are archived in Pangaea (https://doi.org/10.1594/PANGAEA.967716) . The cores are stored in the IODP core repository in Japan (https://www.kochi-core.jp/en/iodp-curation/index.html). Source data for figure 5 are available as a Source Data file. All IODP mission-specific platform data are accessible at http://iodp.pangea.de. CT-images of the whole-round core sections are available at the Fairdata.fi portal: https://doi.org/10.23729/22b512b4-58c0-447d-849d-360c6d645c22.

Research involving human participants, their data, or biological material

Policy information about studies with human participants or human data. See also policy information about sex, gender (identity/presentation), and sexual orientation and race, ethnicity and racism.

Reporting on sex and gender	-
Reporting on race, ethnicity, or other socially relevant groupings	-
Population characteristics	-
Recruitment	-
Ethics oversight	-

Note that full information on the approval of the study protocol must also be provided in the manuscript.

Field-specific reporting

Please select the one below that is the best fit for your research. If you are not sure, read the appropriate sections before making your selection.

☐ Life sciences ☐ Behavioural & social sciences ☒ Ecological, evolutionary & environmental sciences

For a reference copy of the document with all sections, see nature.com/documents/nr-reporting-summary-flat.pdf

Ecological, evolutionary & environmental sciences study design

All studies must disclose on these points even when the disclosure is negative.

Study description	This study is primarily based on sedimentological and ichnological documentation of micro CT-imaged, 20 whole-round core sections (each 17–55 cm long) derived from the trench-basins of Southern, Central and Northern Japan Trench. The data are supported with grain size analyses and C/N data (n=48), which were further combined with previously published analyses from the same cores (n=126; Strasser et al., 2023). Moreover, the data are integrated with previously published core logs, multibeam bathymetric images and seismic sub-bottom profiles (Strasser et al. 2023) to place the studied core sections in sedimentologic and stratigraphic context.
Research sample	CT-images, geochemical C, N, S analyses and grain size distribution data from 20 whole-round core sections from IODP Expedition 386
Sampling strategy	The selected core sections represent all three trench basins, both the main depocenters and the relative bathymetric highs.
Data collection	The data set is based on piston cores collected by The International Ocean Discovery Program (IODP) Expedition 386 from the Japan Trench in 2021
Timing and spatial scale	Coring was carried out in the Japan Trench in April-May 2021
Data exclusions	No data were excluded
Reproducibility	Sample standards were included in geochemical C, N, S and grain size distribution analyses
Randomization	Not applicable, sampling was guided by sedimentological description and recognized facies
Blinding	Not applicable

Did the study involve field work? ☐ Yes ☒ No

Reporting for specific materials, systems and methods

We require information from authors about some types of materials, experimental systems and methods used in many studies. Here, indicate whether each material, system or method listed is relevant to your study. If you are not sure if a list item applies to your research, read the appropriate section before selecting a response.

Materials & experimental systems

n/a	Involved in the study
☒	☐ Antibodies
☒	☐ Eukaryotic cell lines
☒	☐ Palaeontology and archaeology
☒	☐ Animals and other organisms
☒	☐ Clinical data
☒	☐ Dual use research of concern
☒	☐ Plants

Methods

n/a	Involved in the study
☒	☐ ChIP-seq
☒	☐ Flow cytometry
☒	☐ MRI-based neuroimaging

Plants

Seed stocks	-
Novel plant genotypes	-
Authentication	-
