# Supplementary material for: Bioturbation in the hadal zone
Source: Nat Commun. 2025 Feb 18;16:1401. doi: 10.1038/s41467-025-56627-x (PMC11836284; doi:10.1038/s41467-025-56627-x)
Supplement: Supplementary file 1 — Supplementary Information [file 41467_2025_56627_MOESM1_ESM.pdf]

Supplementary File to the article

Bioturbation in the Hadal Zone

Jussi Hovikoski, Joonas J. Virtasalo, Andreas Wetzel, Mishelle Muthre, Michael Strasser, Jean-Noel Proust, Ken Ikehara

This Supplementary file contains:

Supplementary Note 1. Site summary figures
Supplementary Figure 1. M0081 & M0082 – Southern Japan Trench
Supplementary Figure 2. M0083 & M0089 – Central Japan Trench
Supplementary Figure 3. M0084 & M0085 – Northern Japan Trench

Supplementary Note 2. Facies table and an illustrated section of stacked gravity-flow beds
Supplementary Table 1. Facies table
Supplementary Figure 4. Illustrated section of stacked gravity-flow beds

Supplementary Note 3. CT images, facies and grain size distribution plots
Supplementary Table 2. Overview of WR-samples
Supplementary Figure 5. M0084F-1H-19WR22
Supplementary Figure 6. M0084F-1H-20WR23
Supplementary Figure 7. M0084F-1H-21WR24
Supplementary Figure 8. M0085D-1H-2WR25
Supplementary Figure 9. M0085D-1H-3WR26
Supplementary Figure 10. M0085D-1H-3WR27
Supplementary Figure 11. M0083F-1H-10WR16
Supplementary Figure 12. M0083F-1H-16WR17
Supplementary Figure 13. M0089D-1H-3WR34
Supplementary Figure 14. M0089D-1H-5WR35
Supplementary Figure 15. M0089D-1H-5WR36
Supplementary Figure 16. M0081D-1H-5WR1
Supplementary Figure 17. M0081D-1H-6WR2
Supplementary Figure 18. M0081D-1H-10WR3
Supplementary Figure 19. M0081F-1H-7WR4
Supplementary Figure 20. M0082C-1P-1WR6
Supplementary Figure 21. M0082D-1H-2WR7
Supplementary Figure 22. M0082D-1H-3WR8
Supplementary Figure 23. M0082D-1H-4WR9
Supplementary Figure 24. M0082D-1H-5WR10

Supplementary Note 4. Incipient trace fossil table and plates
Supplementary Table 3. Incipient trace fossil table
Supplementary Figure 25. Incipient trace fossil images

Supplementary Note 5. Additional statistical graphs
Supplementary Figure 26. Plots of measured bioturbation properties in the trench basins

Supplementary Refrerences

Supplementary Note 1

Site summary figures

This section presents site summary figures for the studied trench basins:

Supplementary Figure 1. M0081 & M0082 – Southern Japan Trench
Supplementary Figure 2. M0083 & M0089 – Central Japan Trench
Supplementary Figure 3. M0084 & M0085 – Northern Japan Trench

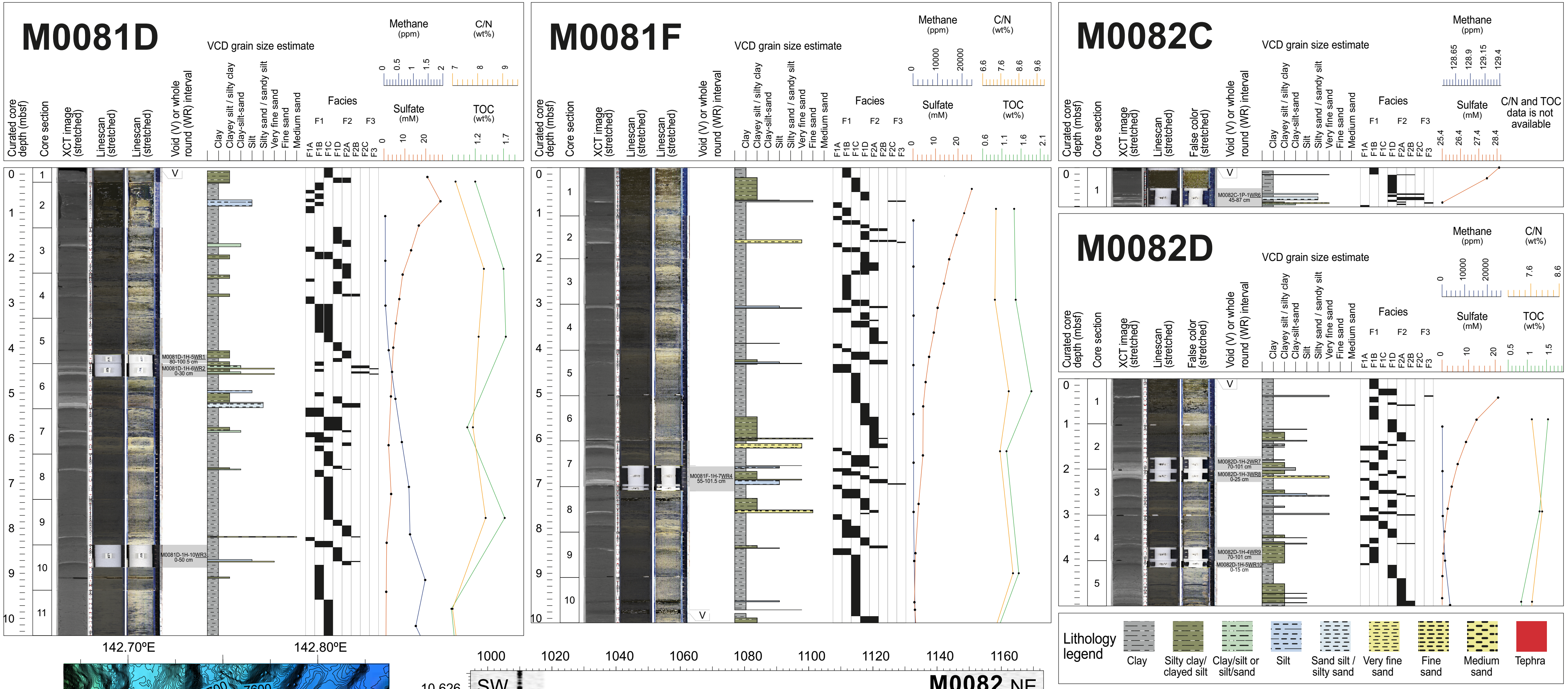

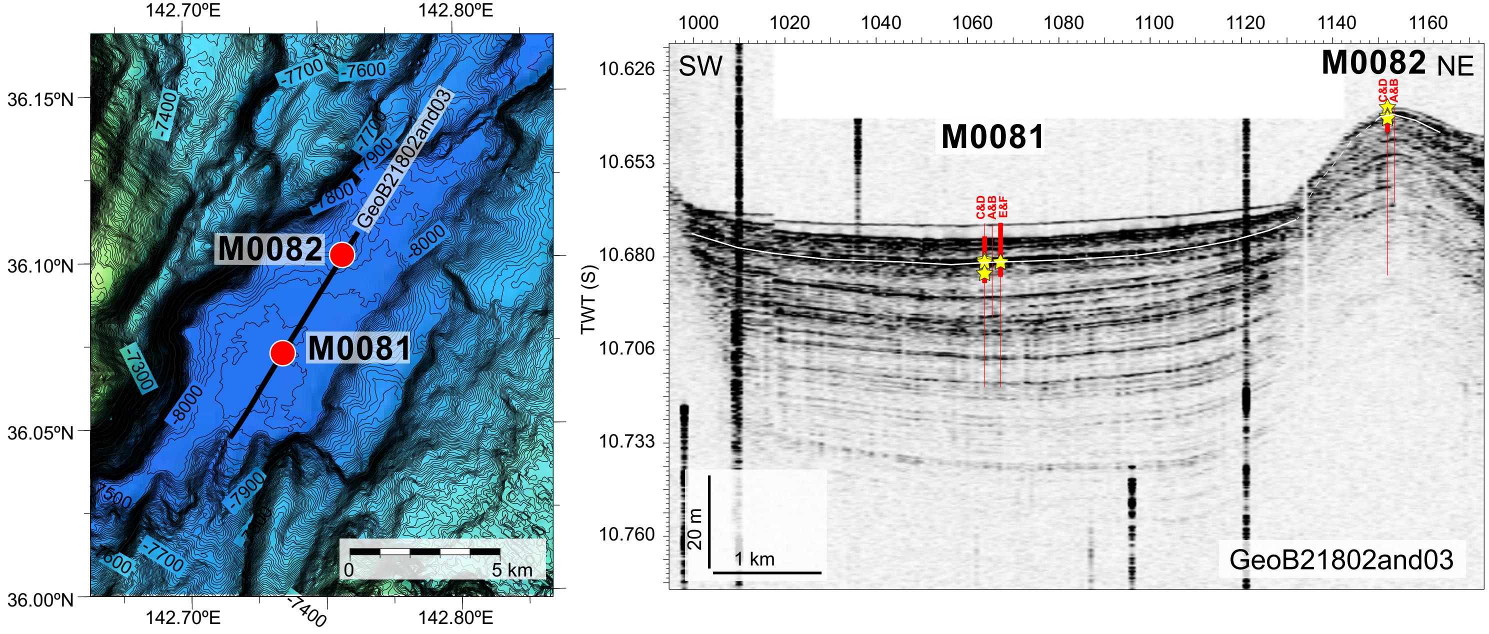

Supplementary Figure 1: Location, trench basin physiography and basin-fill sequence characteristics of Sites M0081 and M0082.
Left: high-resolution bathymetric map with 5 m contours, site locations, and track line of high-resolution sub-bottom profile show on the right.
Right: site survey sub-bottom profile showing depths (assuming 1500 m/s P-wave velocity) of holes cored (fine red lines), intervals presented as lithostratigraphic logs (thick red lines) and the locations of whole-round samples analysed in this study (yellow stars). White line shows the lateral extend and correlation of stratigrapic marker reflector used as the tie point for stratigraphic correlation between the sites[1].

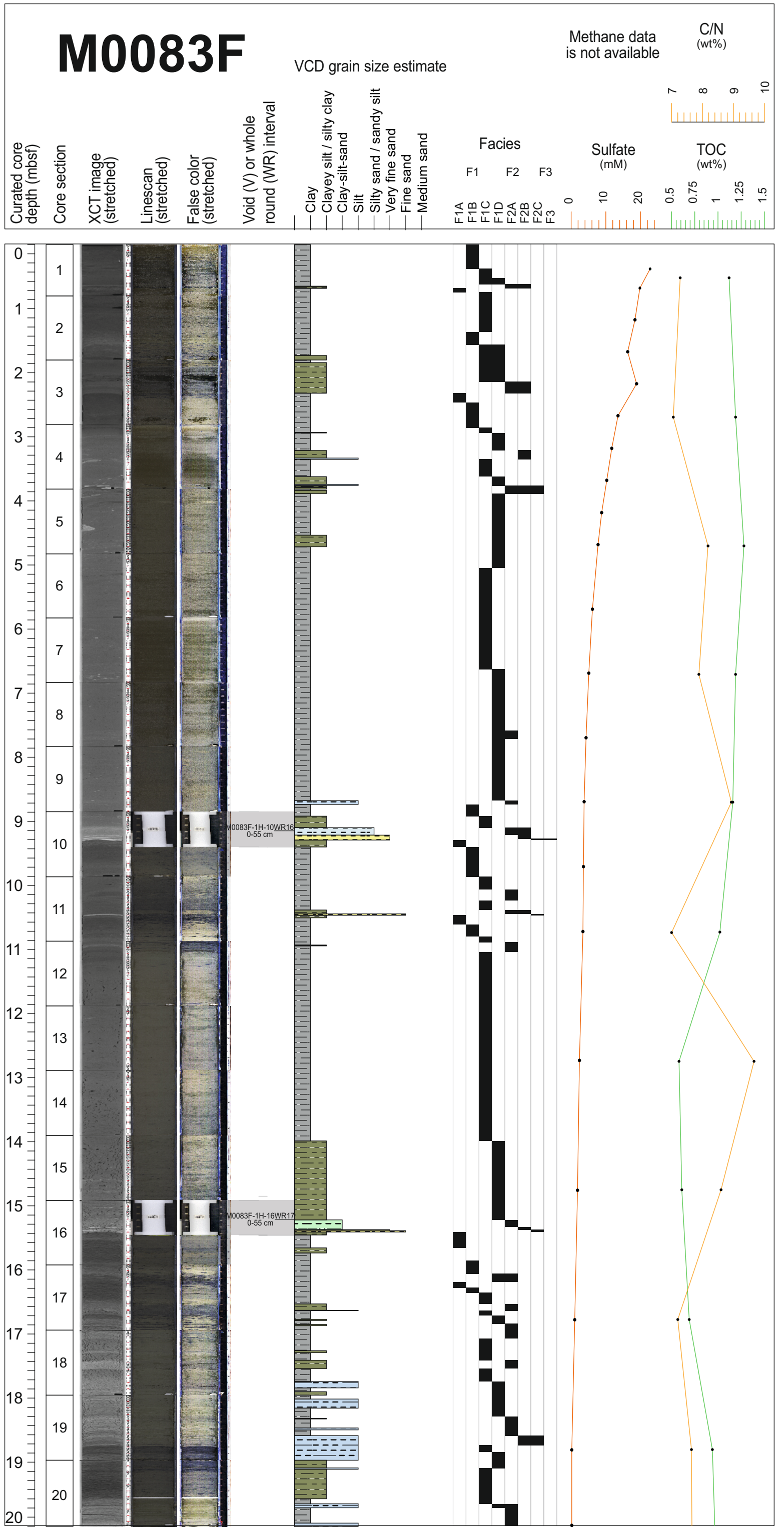

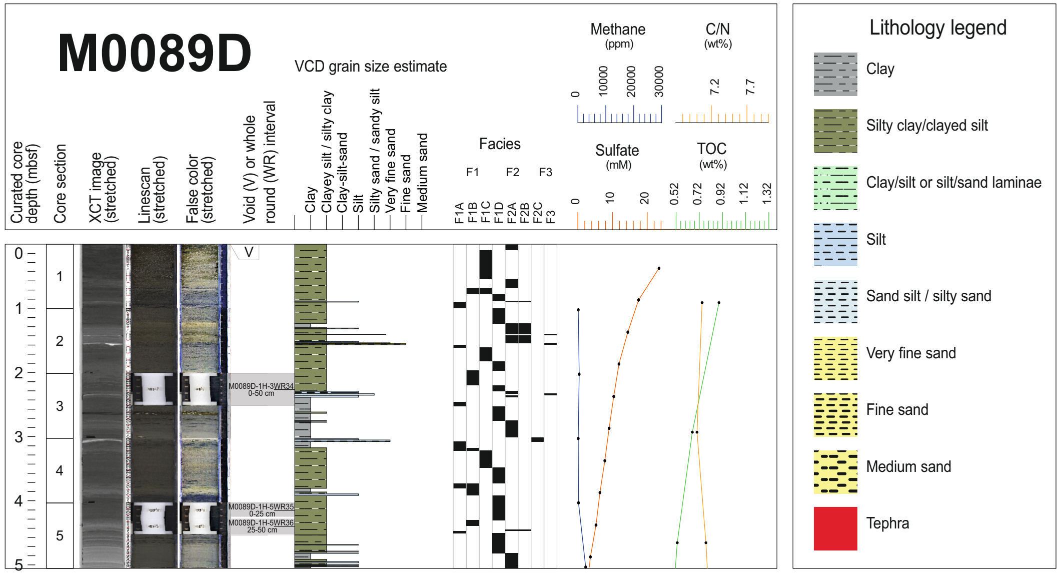

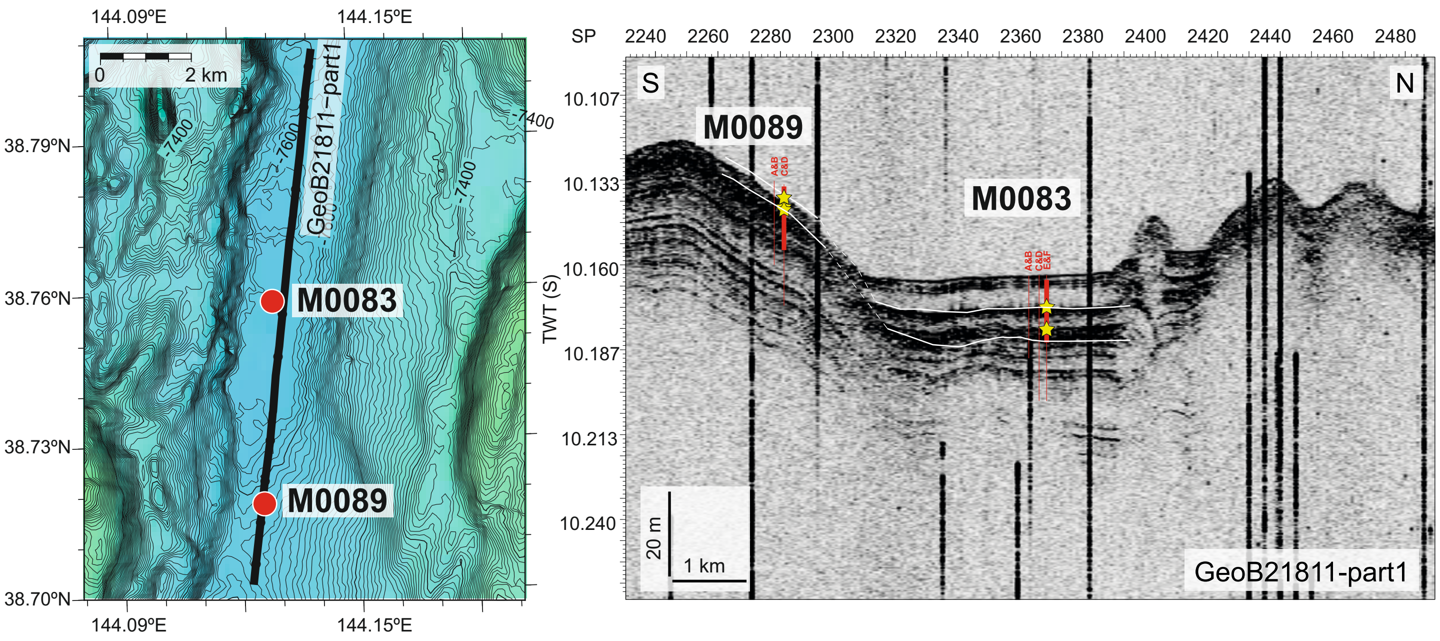

Supplementary Figure 2: Location, trench basin physiography and basin-fill sequence characteristics of Sites M0083 and M0089. Left: high-resolution bathymetric map with 5 m contours, site locations, and track line of high-resolution sub-bottom profile show on the right.
Right: site survey sub-bottom profile showing depths (assuming 1500 m/s P-wave velocity) of holes cored (fine red lines), intervals presented as lithostratigraphic logs (thick red lines) and the locations of whole-round samples analysed in this study (yellow stars). White line shows the lateral extend and correlation of stratigrapic marker reflector used as the tie point for stratigraphic correlation between the sites[1].

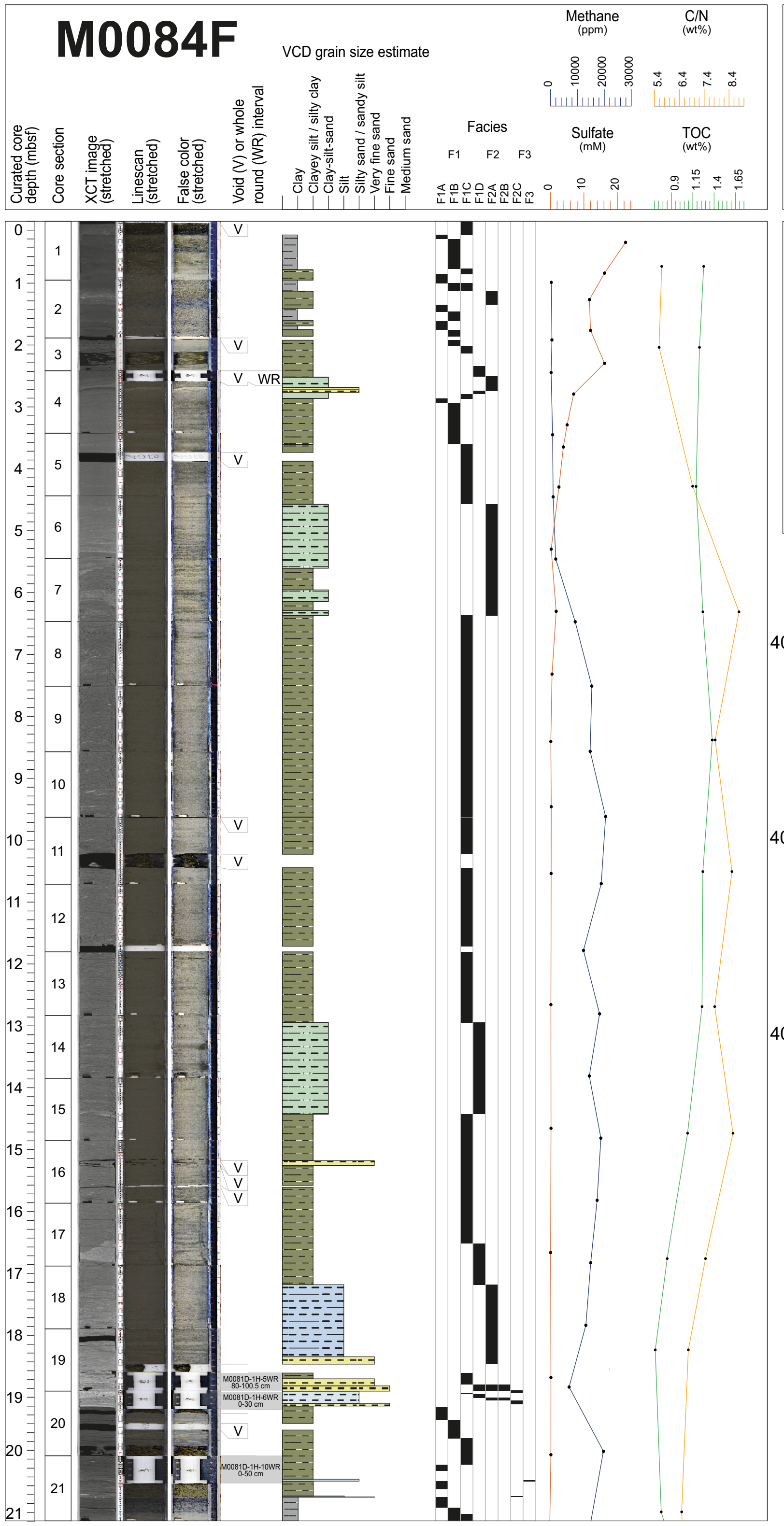

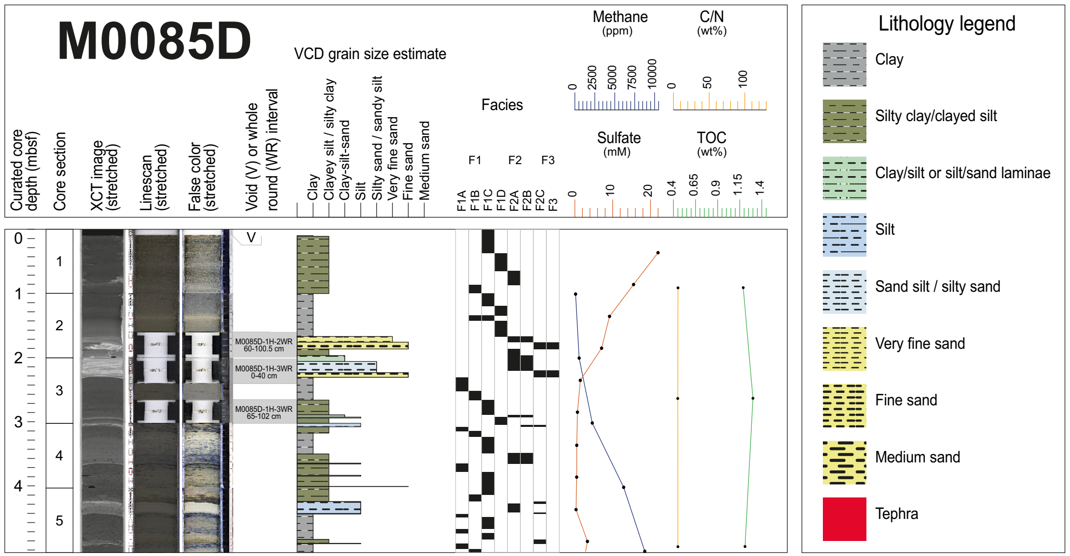

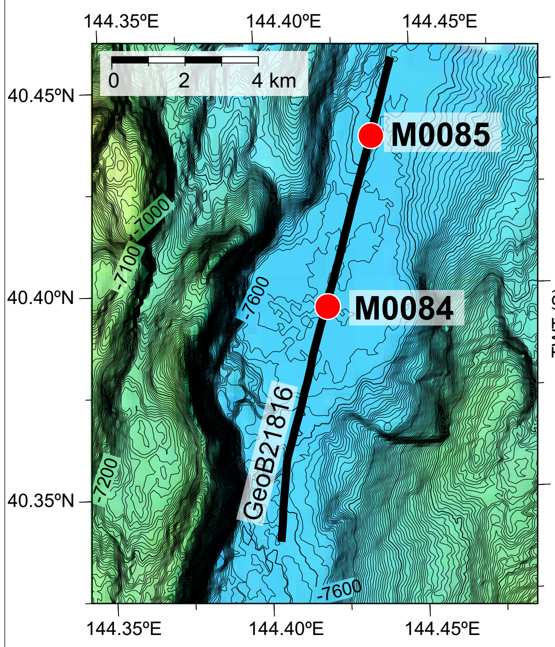

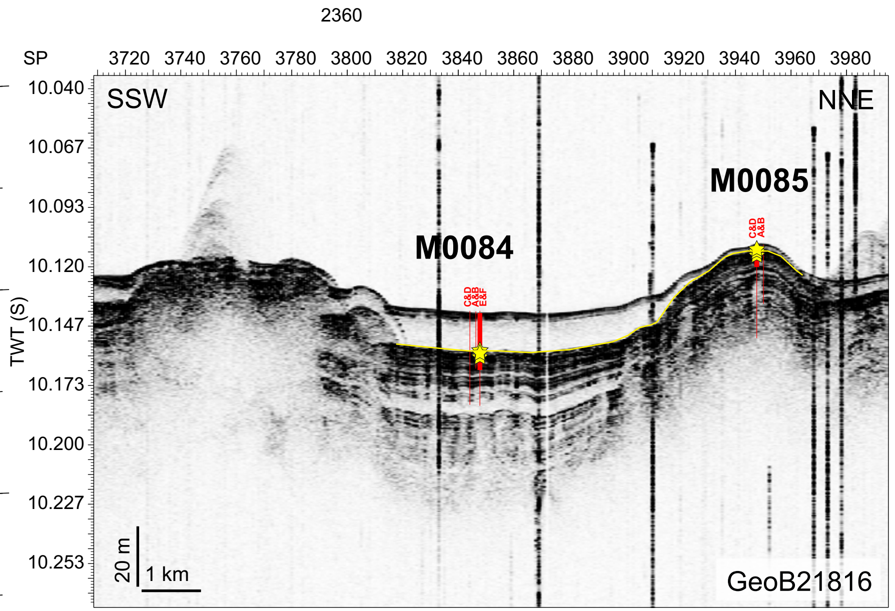

Supplementary Figure 3: Location, trench basin physiography and basin-fill sequence characteristics of Sites M0084 and M0085. Left: high-resolution bathymetric map with 5 m contours, site locations, and track line of high-resolution sub-bottom profile show on the right.
Right: site survey sub-bottom profile showing depths (assuming 1500 m/s P-wave velocity) of holes cored (fine red lines), intervals presented as lithostratigraphic logs (thick red lines) and the locations of whole-round samples analysed in this study (yellow stars). White line shows the lateral extend and correlation of stratigrapic marker reflector used as the tie point for stratigraphic correlation between the sites[1].

Supplementary Note 2

Supplementary Table 1. Facies Table

**Facies**	**Occurrence**	**Sedimentology**	**Interpretation**
F1: Mud			
F1A: Bioturbated mud with pyrite	Common facies in all trench-basins. F1A occurs above F1B or F1C. It is sharply or erosionally overlain by F3.	F1A consists of surface or a mm-thick narrow zone in the top of F1B or F1C occurrences, from which bioturbation descends into underlying sediments, leading to mm- to dm-scale occurrences of bioturbated mud. Preservation of the unit can be affected by an erosional contact above. The recognition of the facies is based on trace fossils and their high-density burrow fills (e.g., pyrite or greigite). Trace fossils include slender helicoidal traces (Gy), Ch-group traces (Pi), Tr and/or Zo. The burrows typically cross-cut trace fossil fabric of F1B or descend into F1C, reaching variable depths.	Hemipelagic–pelagic, dilute suspension settling or clear water conditions. The sediments are dewatered (soft–stiff ground) prior to colonization.
F1B: Burrow mottled mud	Common facies in all trench-basins. F1B occurs above F2 and below F1A.	F1B consists of cm- to dm-scale occurrences of bioturbated mud. Bioturbation intensity is typically high (BI4–6) but fluctuates (locally down to BI1–2). Locally visible are palimpsest laminae and normal grading. F1B is typically bioturbated by Ph, Ne, and Rosselichnid traces such as Ar.	Colonization following the main part (traction phase) of a gravity flow event. F1B broadly corresponds with division T8 of the Stow sequence but differs from it by intense bioturbation forming up to tens of cm thick intervals.
F1C: Structureless mud	Recurring facies particularly in the Southern and Central Japan Trench. F1C overlies F2 and can be overlain by other F1 sub-facies.	F1C comprises cm- to dm-scale structureless mud intervals, which appear ungraded in the micro CT-images. It consists of silty clay or clayey silt with sporadic sand grains. Locally, the structureless mud intervals contain loading structures such as pseudonodules. The pseudonodules are silt/sand-filled rounded masses that descend into the mud-matrix from above. These pseudonodules are from a few mm to 4 cm wide and connected upward to a vertical irregular silt or sand trail, which can be up to 20 cm long.	Gravity flow fluid mud and suspension settling (densite mud[2]). Locally, subsequent gravity flow deposits can descend into the fluid-like sediment (soup ground) forming detached pseudonodular structures. Although most occurrences of deformation are syn-sedimentary (bed or lamina restricted), post-depositional liquification cannot be always ruled out. F1C corresponds to division T7 in the Stow sequence[3].
F1D: Graded mud	Recurring facies occurring particularly in the Southern and Northern Japan Trench. F1D overlies F1C or is interbedded with F2. It can form top of the bed or F1B overlies it.	F1D consists of normally graded mud or stacked normally graded structureless mud bands. Individual bands are 0.5–1 cm thick, whereas the sub-facies forms upto a few cm thick occurrences.<br>Small scale soft-sedimentary structures are common.	Differential settling of silt and clay from the suspension cloud. When F1D overlies F1C, the sub-facies probably reflects decreasing sediment concentration at the end of the flow event. F1D bears similarities with the division T6 in the Stow sequence, but forms commonly repeated bands and can be positioned in the top of a bed.

Continues...

...continued

Facies	Occurrence	Sedimentology	Interpretation
F2: Heterolithic mud (clay-silt)			
F2: Laminated clay and silt	Common facies in all trench-basins. F2 occurs gradationally above F3 or is interbedded with it. F2 is commonly overlain by F1B or F1C	F2 is broad facies type consisting of heterolithic lamination formed dominantly by silt and clay, locally with minor sand component. Bulk lithology is commonly clayey silt. The facies occurrences are typically 2–10 cm thick. F2 can be divided into following sub-facies:<br>- F2A: Parallel laminated silt and clay, which can show intervals of inverse to normal grading. Individual laminae are sub-mm thick.<br>- F2B: Cross-laminated or convergent laminated silt and clay, with laminae pinch outs.<br>- F2C: Convoluted structureless mud, forming laminae scale occurrences.<br><br>Finally, F2 is typically unbioturbated, but contains rare syn-depositional Rh and escape structures, and can be burrowed by traces descending from the overlying F1B.	Deposits of slow-moving traction-sediment suspensions. Contains locally mud-floccule ripples (F2B). Variable sediment concentration leads to alternating turbulence, which is locally damped. F2A is similar to divisions T3–T4, whereas F2B corresponds to division T2 in the Stow sequence. Similarly, F2C is similar to division T1[3].
F3: Silt-(sand)			
F3: Ripple-cross lamination	Recurring facies type. F3 occurs typically below F2 forming the base of an event bed.	F3 consists of erosional or sharp-based ripple cross-laminated silt–sand. It forms locally "fading ripples"[4]). Ripple height is typically 0.4–2 cm. F3 is normally unbioturbated.	Lower flow regime traction deposits under turbulent flow (cf. T0 of Stow sequence).

Abbreviations

Ar–*Artichnus*, Ch–*Chondrites*, Gy–*Gyrolithes*, Ne–*Nereites*, Ph–*Phycosiphon*, Pi–*Pilichnus*, Rh–*Rhizocorallium*, Tr–*Trichichnus*, Zo–*Zoophycus*; BI–bioturbation intensity

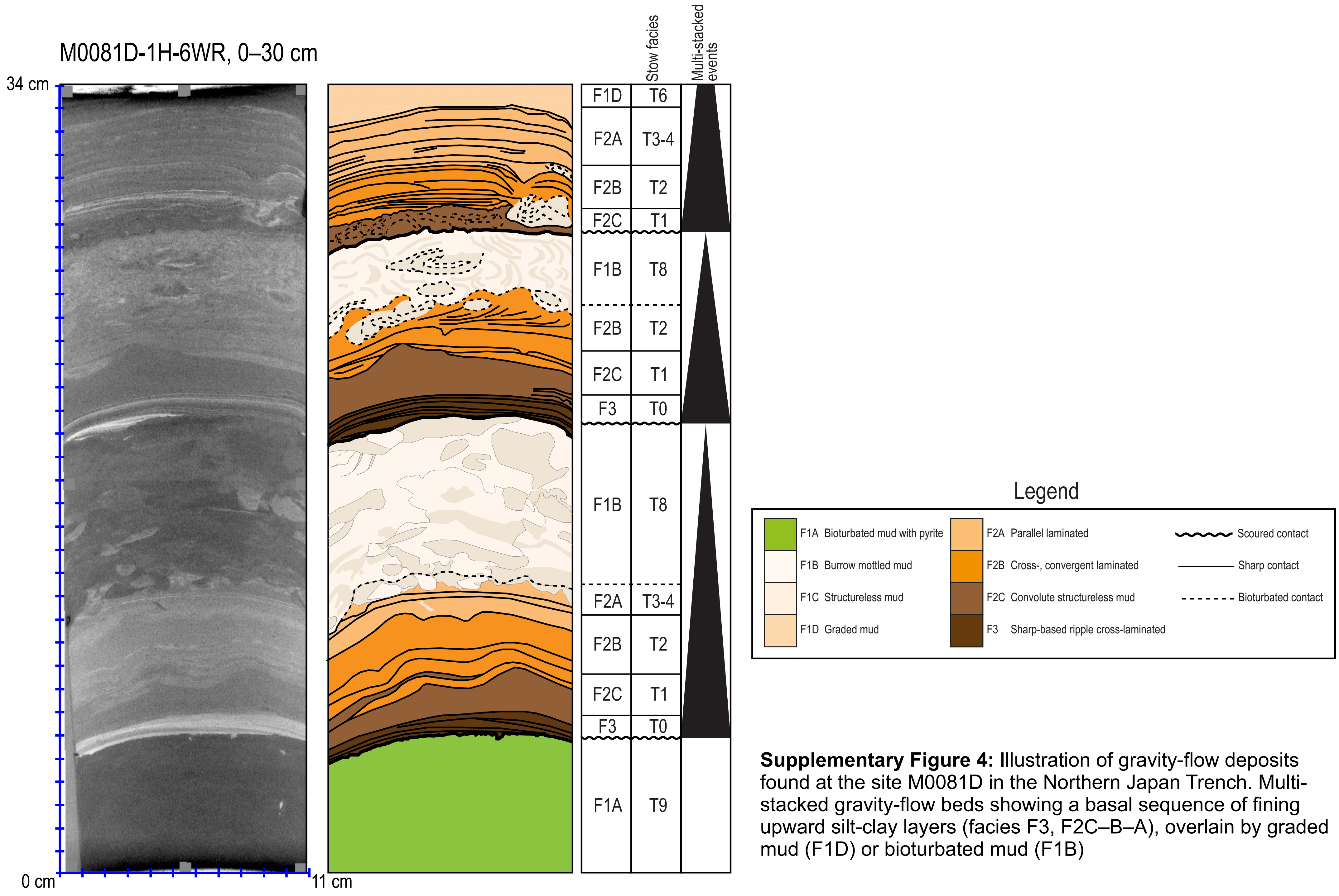

Supplementary Figure 4: Illustration of gravity-flow deposits found at the site M0081D in the Northern Japan Trench. Multi-stacked gravity-flow beds showing a basal sequence of fining upward silt-clay layers (facies F3, F2C–B–A), overlain by graded mud (F1D) or bioturbated mud (F1B)

Supplementary Note 3

CT images, facies and grain size and S, C, N distribution plots

Supplementary Figures 5–24 illustrate CT images, facies and grain size distribution and sulphur, carbon and nitrogen content plots of the whole-round samples listed in the Supplementary Table 3.
For the original grain size and C, N and S data, see: Virtasalo, J.J., Hovikoski, J., 2024. Multielement and grain-size data of sediment cores from the IODP Expedition 386 Japan Trench Paleoseismology. PANGAEA, doi:10.1594/PANGAEA.967716

Supplementary Table 2. Overview of the WR-samples. The WR codes in the article refer to the WR codes underlined in the table. The top and bottom depths refer to the depths in the respective core sections.

Core section	Top depth (cm)	Bottom depth (cm)	Sample length (cm)	Sub-basin
M0084F-1H-19WR22	70.0	100.5	30.5	Northern Japan Trench
M0084F-1H-20WR23	0.0	30.0	30.0	
M0084F-1H-21WR24	0.0	45.0	45.0	
M0085D-1H-2WR25	60.0	100.5	40.5	
M0085D-1H-3WR26	0.0	40.0	40.0	
M0085D-1H-3WR27	65.0	102.0	37.0	
M0083F-1H-10WR16	0.0	55.0	55.0	Central Japan Trench
M0083F-1H-16WR17	0.0	55.0	55.0	
M0089D-1H-3WR34	0.0	50.0	50.0	
M0089D-1H-5WR35	0.0	25.0	25.0	
M0089D-1H-5WR36	25.0	50.0	25.0	
M0081D-1H-5WR1	80.0	100.5	20.5	Southern Japan Trench
M0081D-1H-6WR2	0.0	30.0	30.0	
M0081D-1H-10WR3	0.0	50.0	50.0	
M0081F-1H-7WR4	55.0	101.5	46.5	
M0082C-1P-1WR6	45.0	87.0	42.0	
M0082D-1H-2WR7	70.0	101.0	31.0	
M0082D-1H-3WR8	0.0	25.0	25.0	
M0082D-1H-4WR9	70.0	101.0	31.0	
M0082D-1H-5WR10	0.0	15.0	15.0	

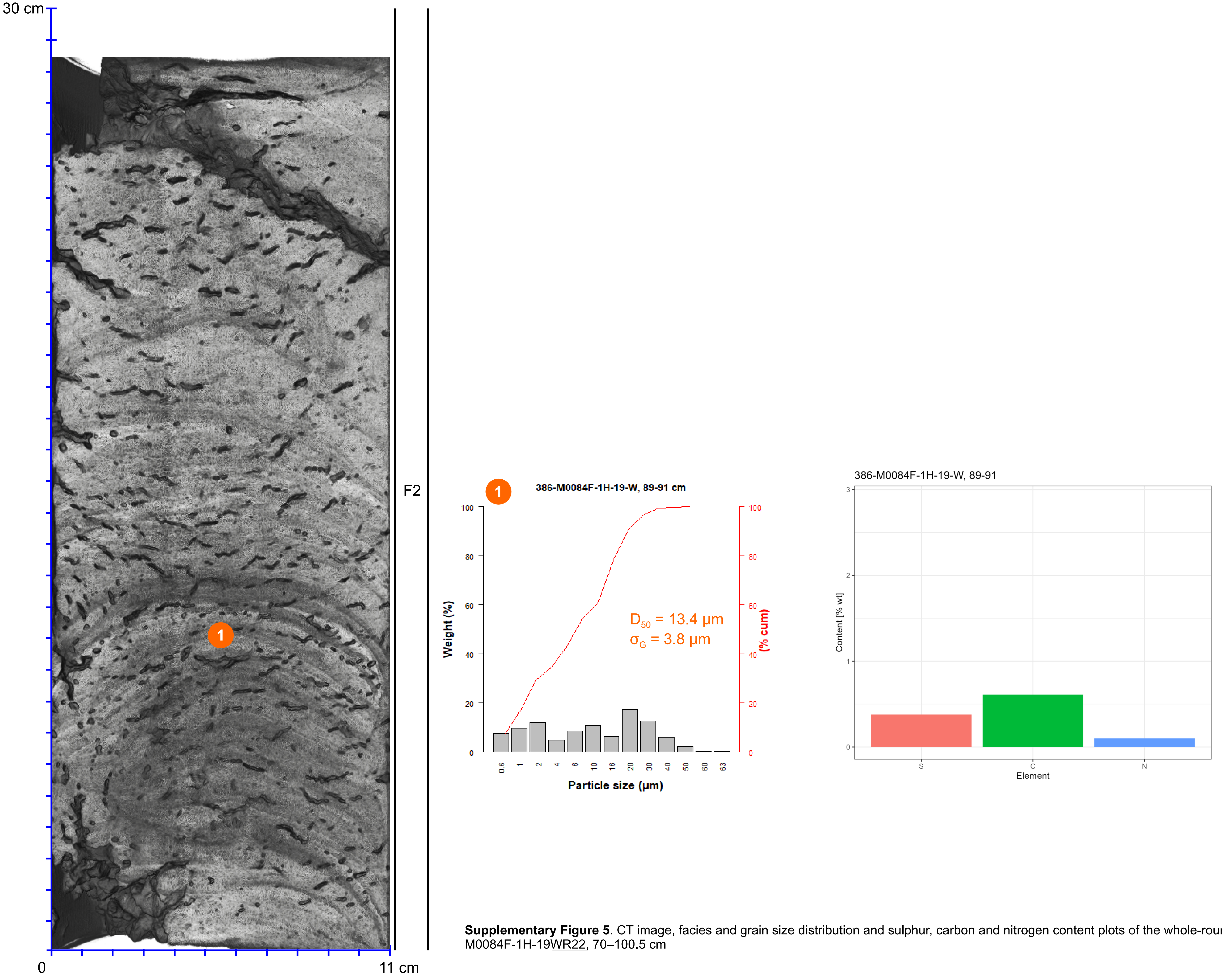

Supplementary Figure 5. CT image, facies and grain size distribution and sulphur, carbon and nitrogen content plots of the whole-round sample M0084F-1H-19WR22, 70–100.5 cm

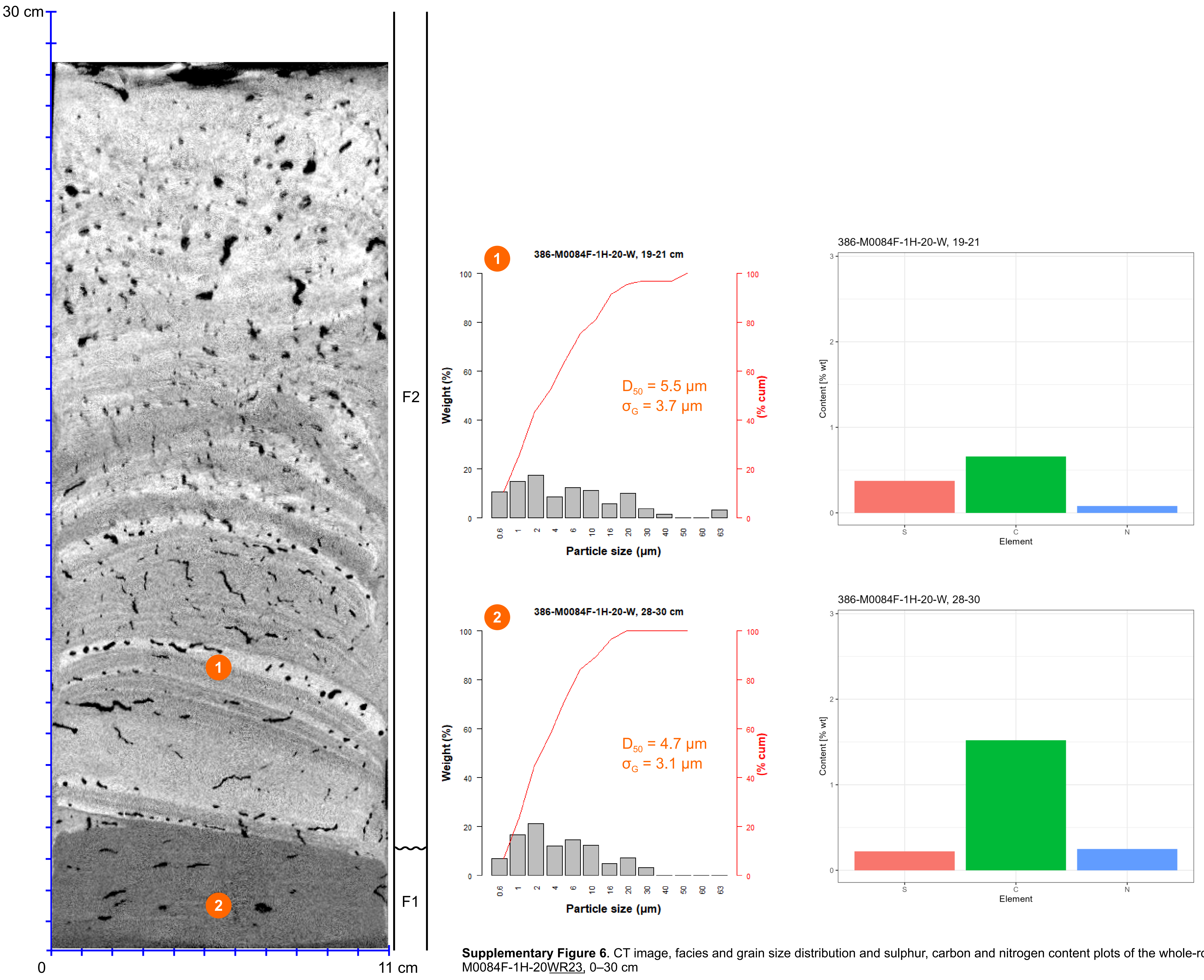

Supplementary Figure 6. CT image, facies and grain size distribution and sulphur, carbon and nitrogen content plots of the whole-round sample M0084F-1H-20WR23, 0–30 cm

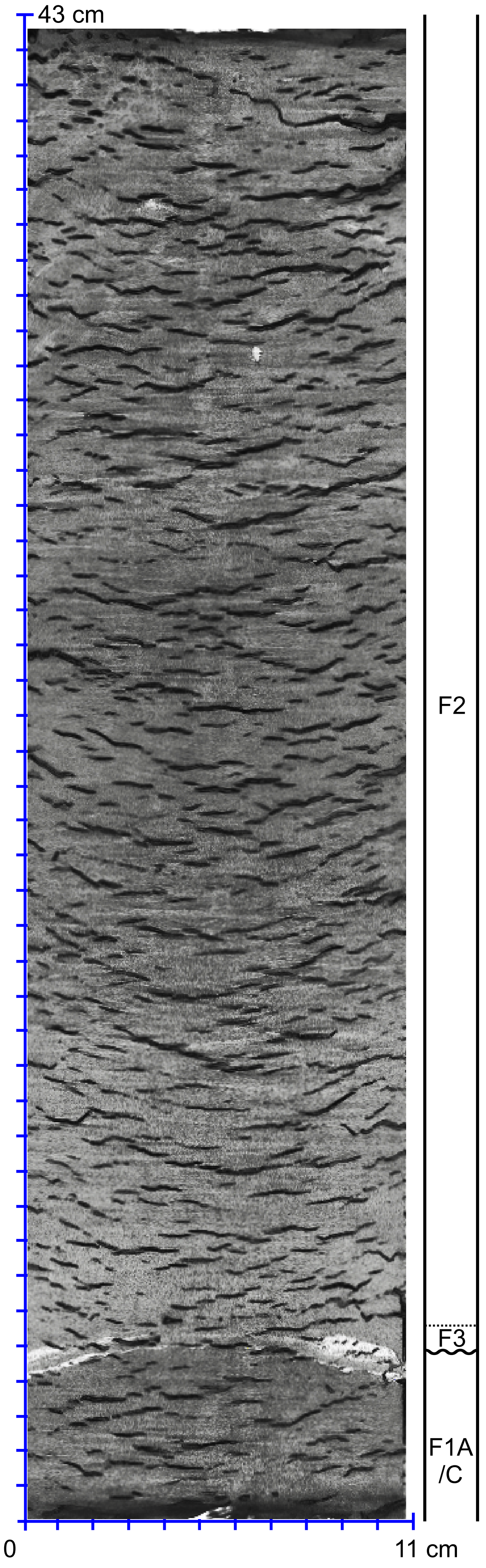

Supplementary Figure 7. CT image and facies of the whole-round sample M0084F-1H-21WR24, 0–45 cm

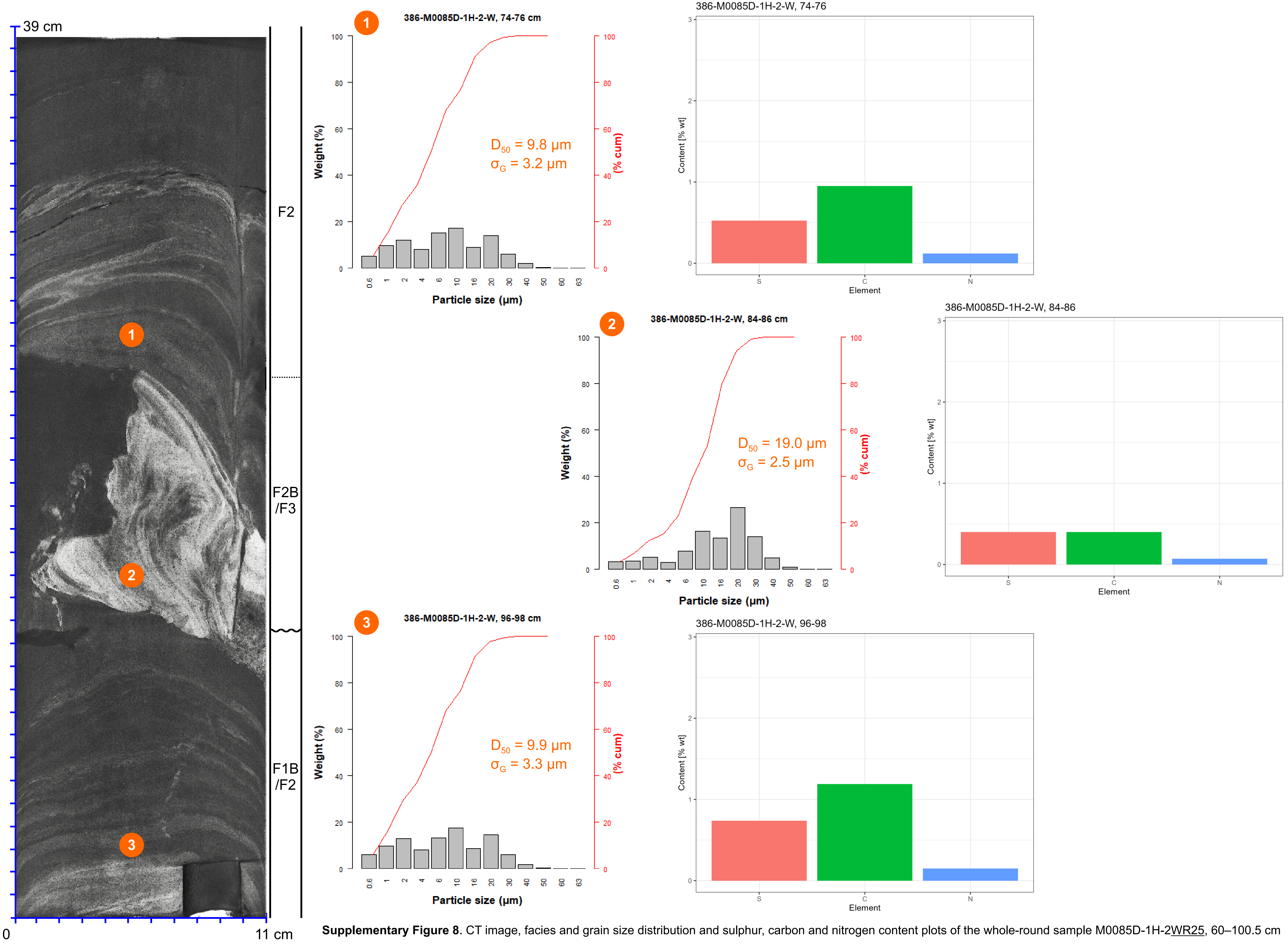

Supplementary Figure 8. CT image, facies and grain size distribution and sulphur, carbon and nitrogen content plots of the whole-round sample M0085D-1H-2WR25, 60–100.5 cm

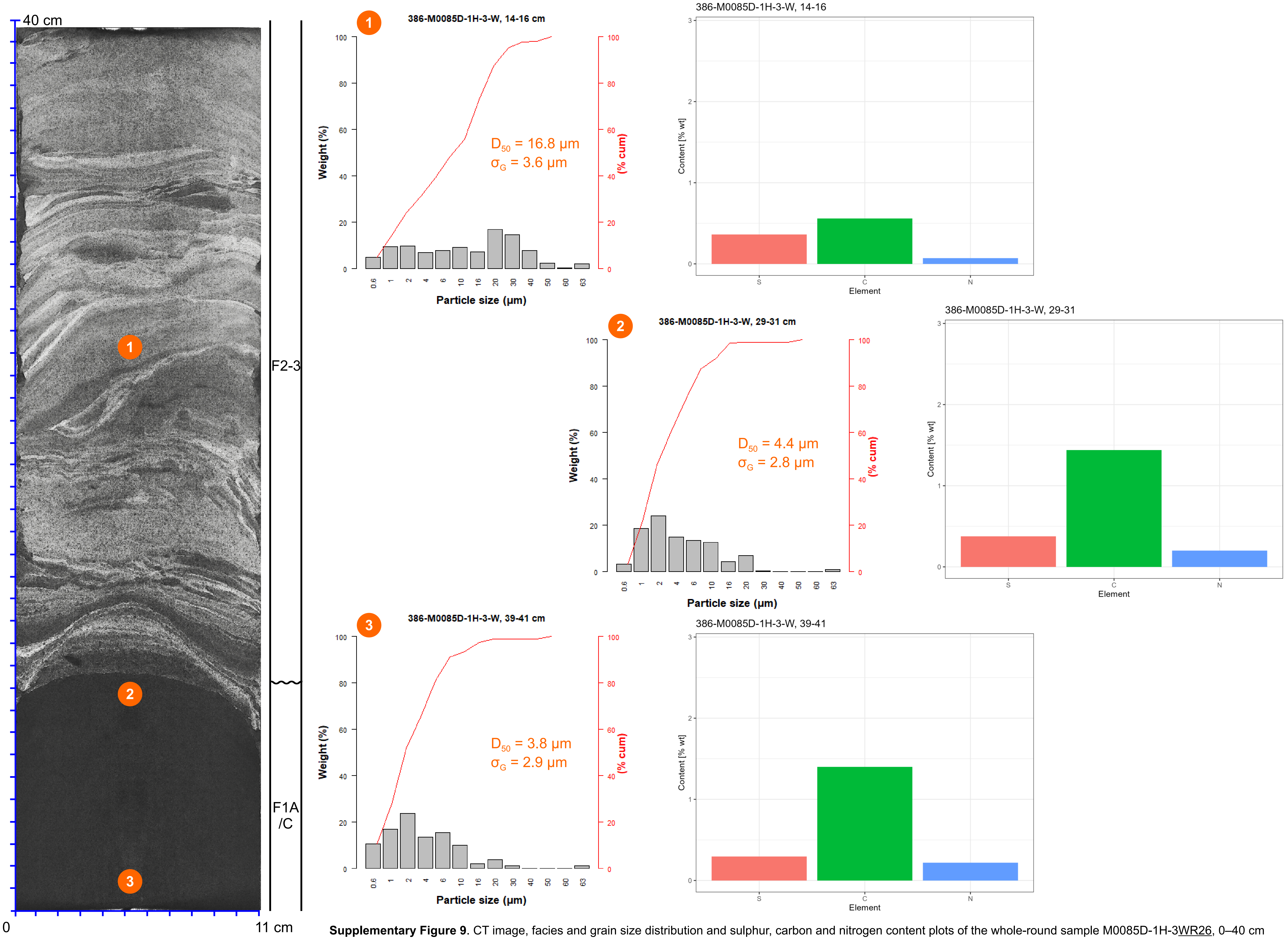

Supplementary Figure 9. CT image, facies and grain size distribution and sulphur, carbon and nitrogen content plots of the whole-round sample M0085D-1H-3WR26, 0–40 cm

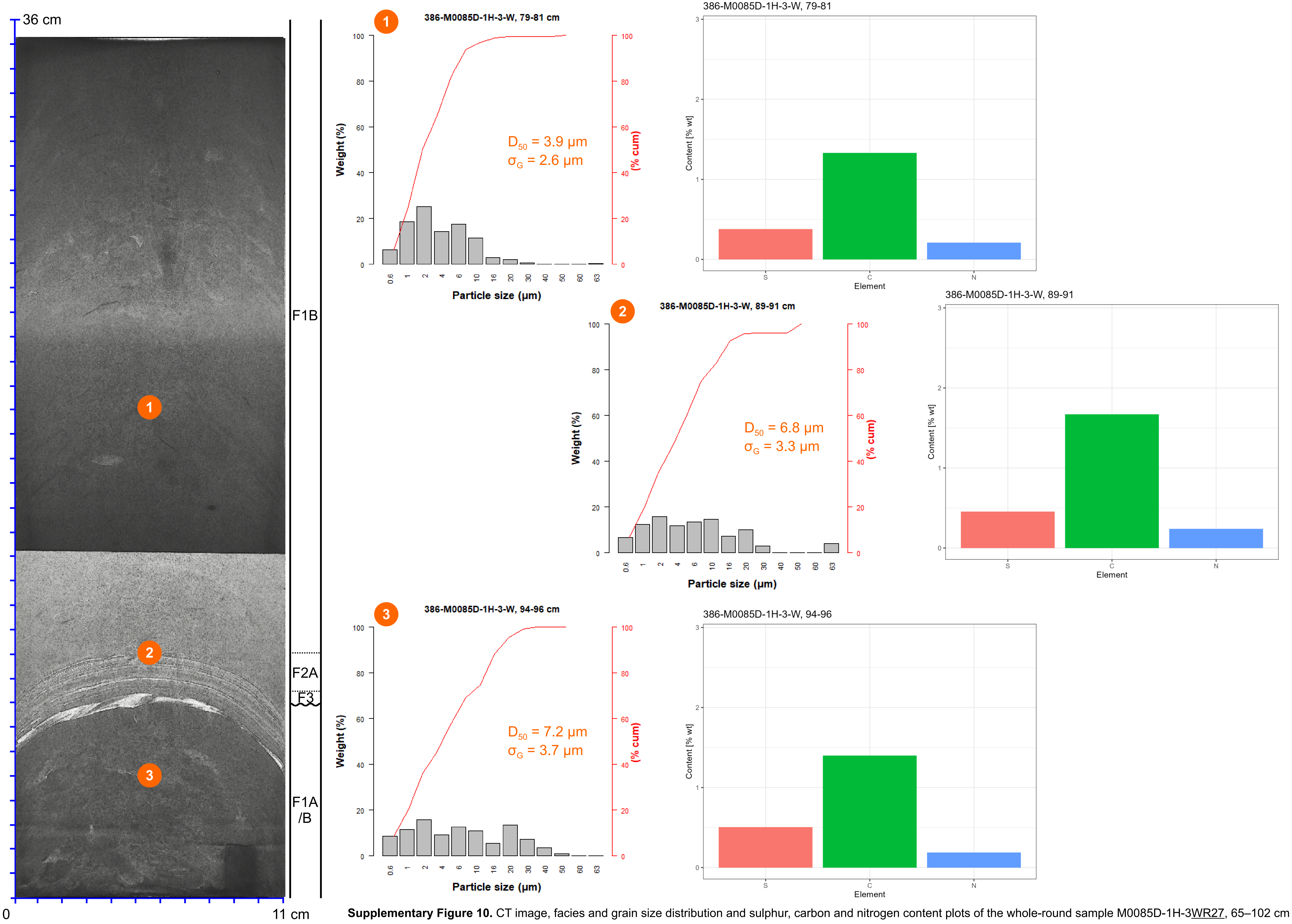

Supplementary Figure 10. CT image, facies and grain size distribution and sulphur, carbon and nitrogen content plots of the whole-round sample M0085D-1H-3WR27, 65–102 cm

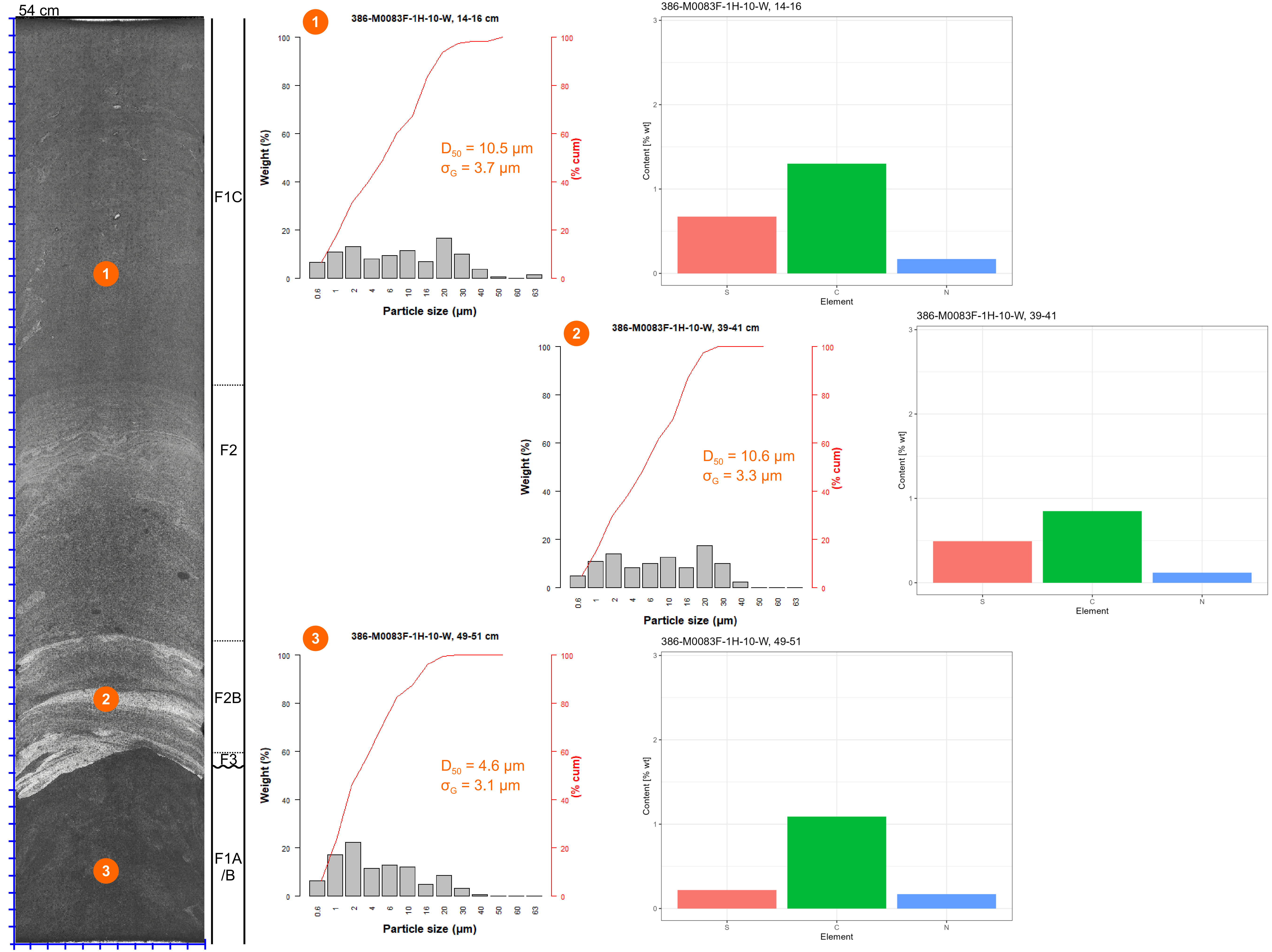

Supplementary Figure 11. CT image, facies and grain size distribution and sulphur, carbon and nitrogen content plots of the whole-round sample M0083F-1H-10WR16, 0–55 cm

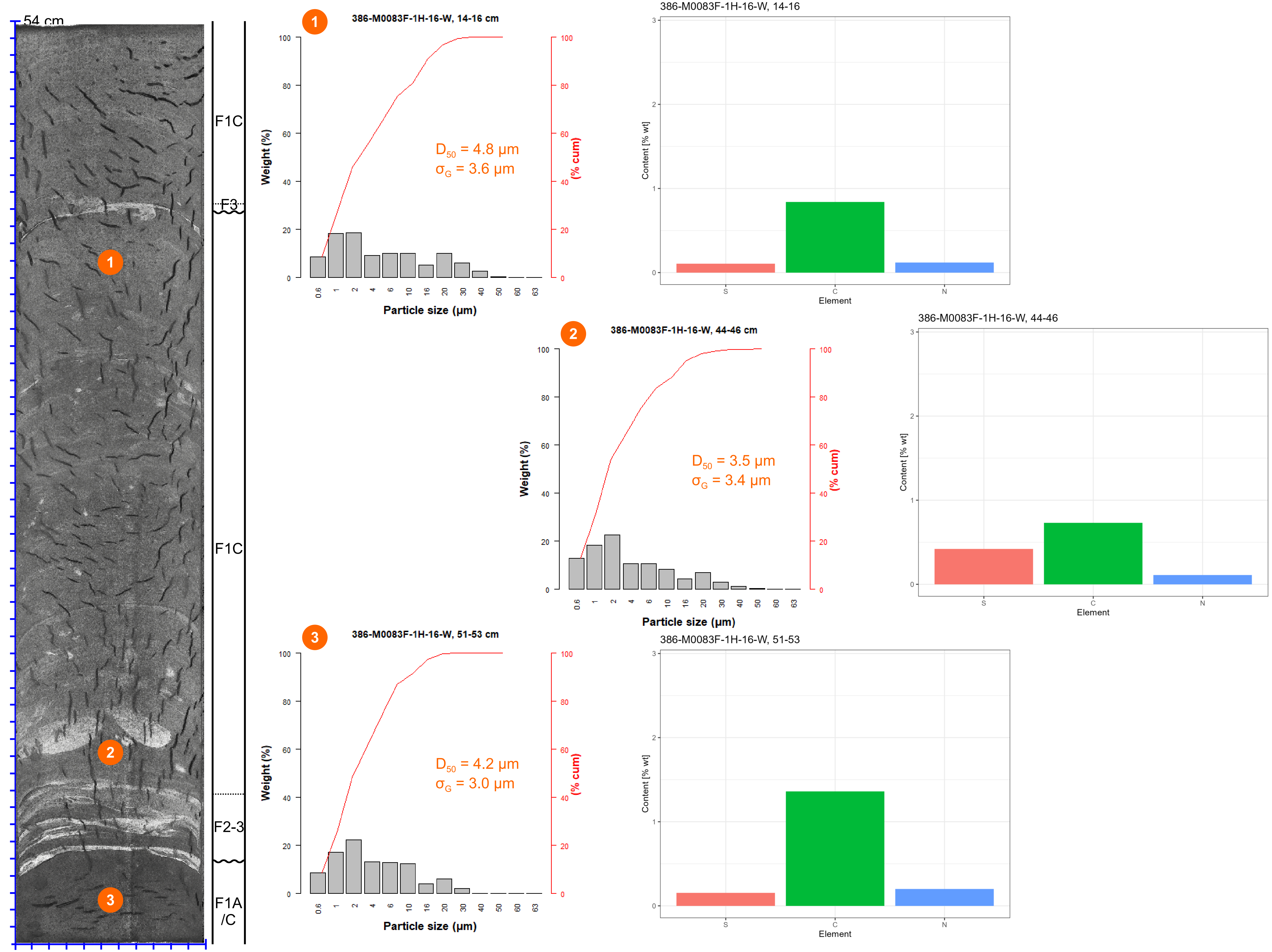

Supplementary Figure 12. CT image, facies and grain size distribution and sulphur, carbon and nitrogen content plots of the whole-round sample M0083F-1H-16WR17, 0–55 cm

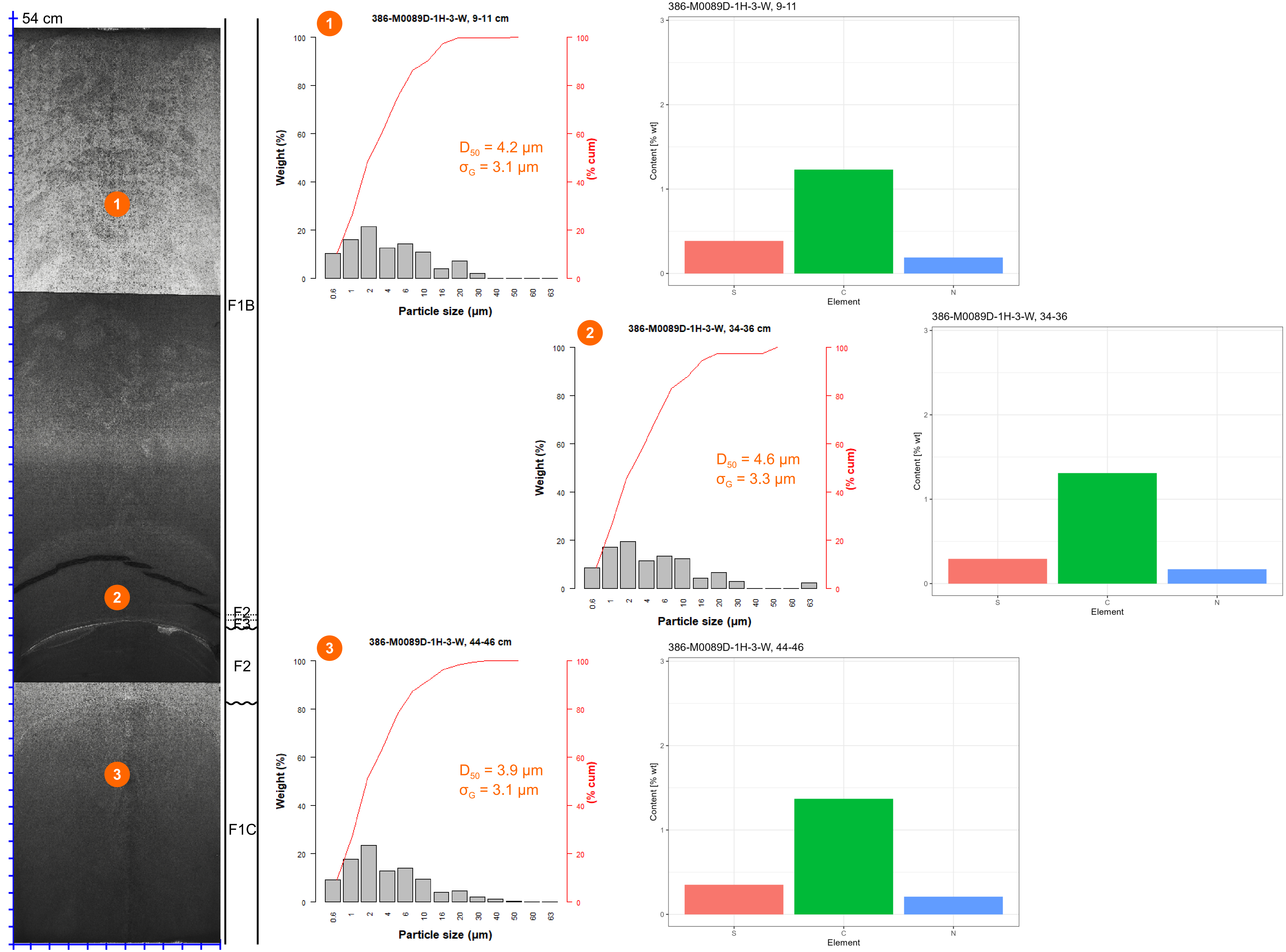

Supplementary Figure 13. CT image, facies and grain size distribution and sulphur, carbon and nitrogen content plots of the whole-round sample M0089D-1H-3WR34, 0–50 cm

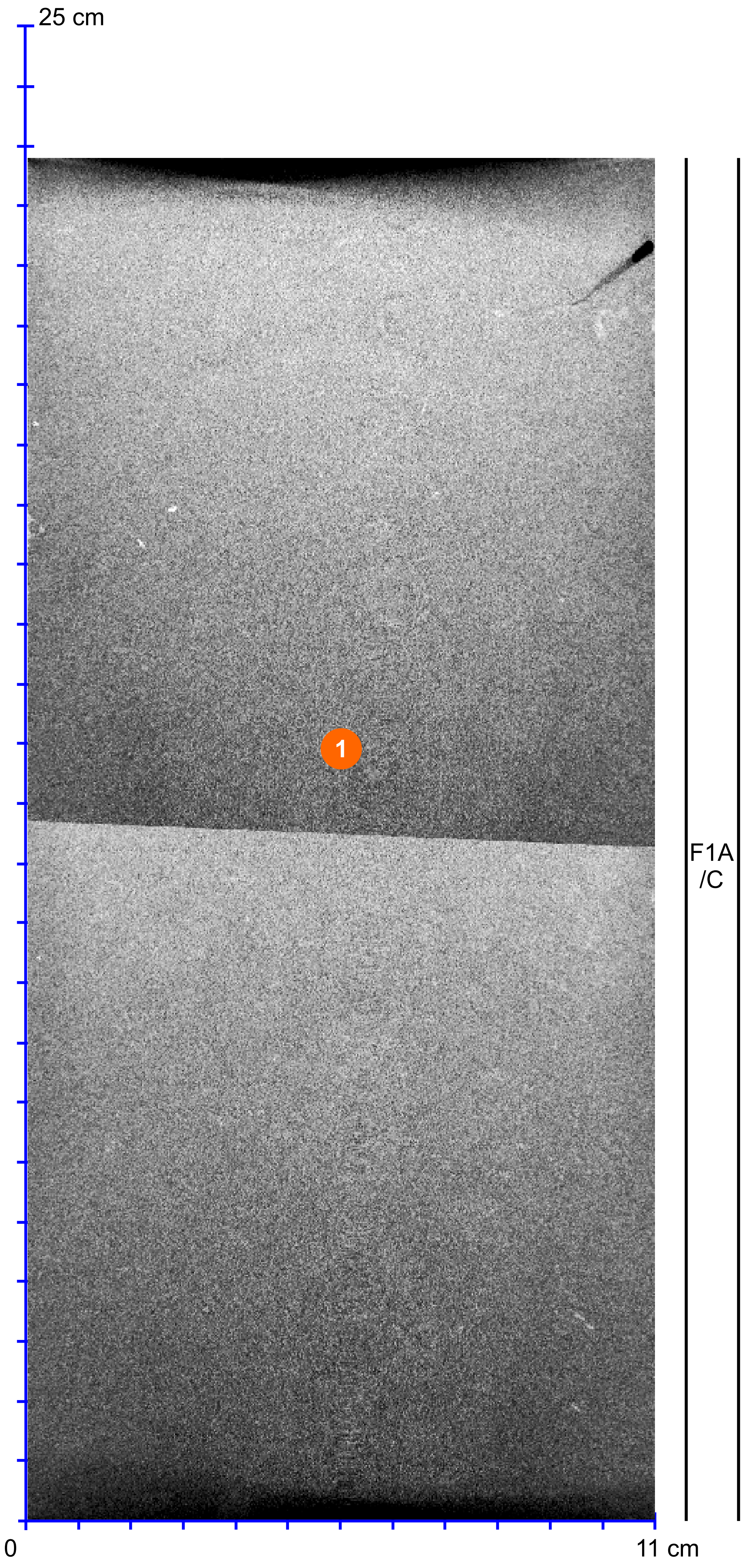

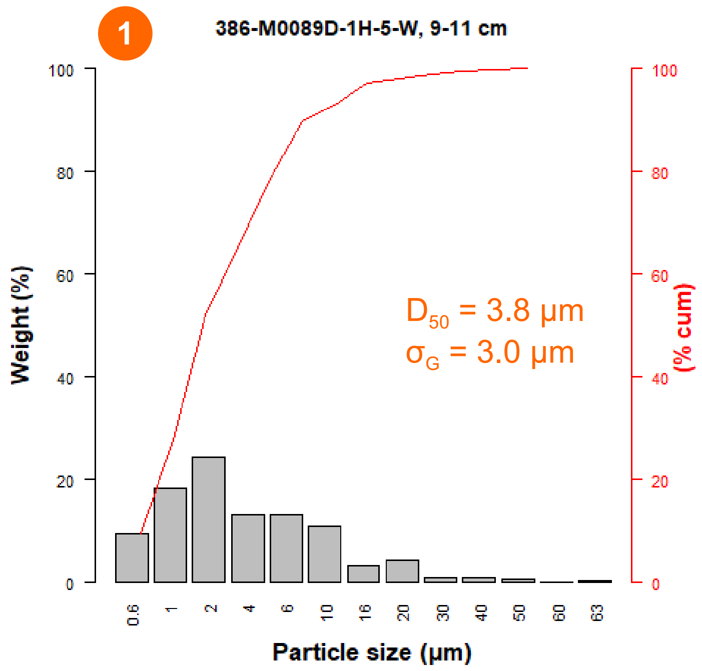

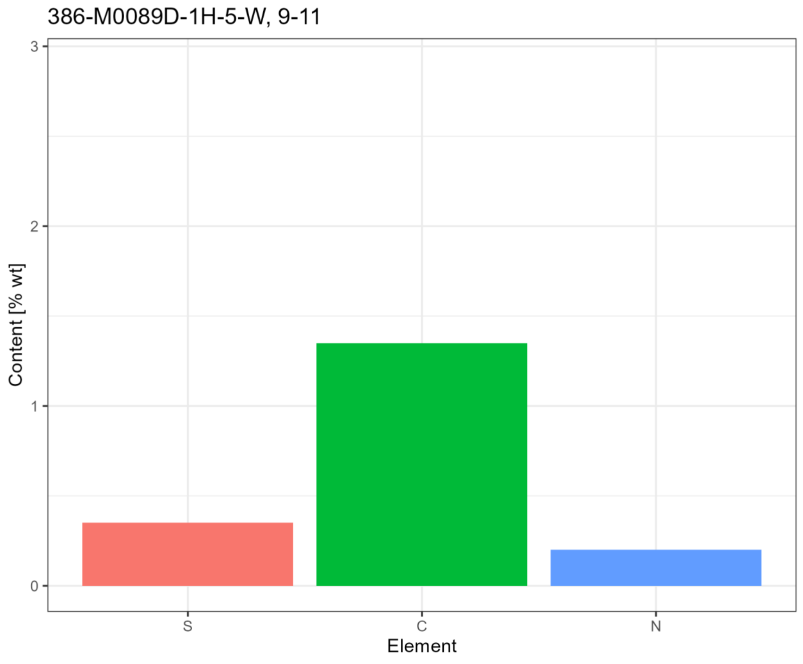

Supplementary Figure 14. CT image, facies and grain size distribution and sulphur, carbon and nitrogen content plots of the whole-round sample M0089D-1H-5WR35, 0–25 cm

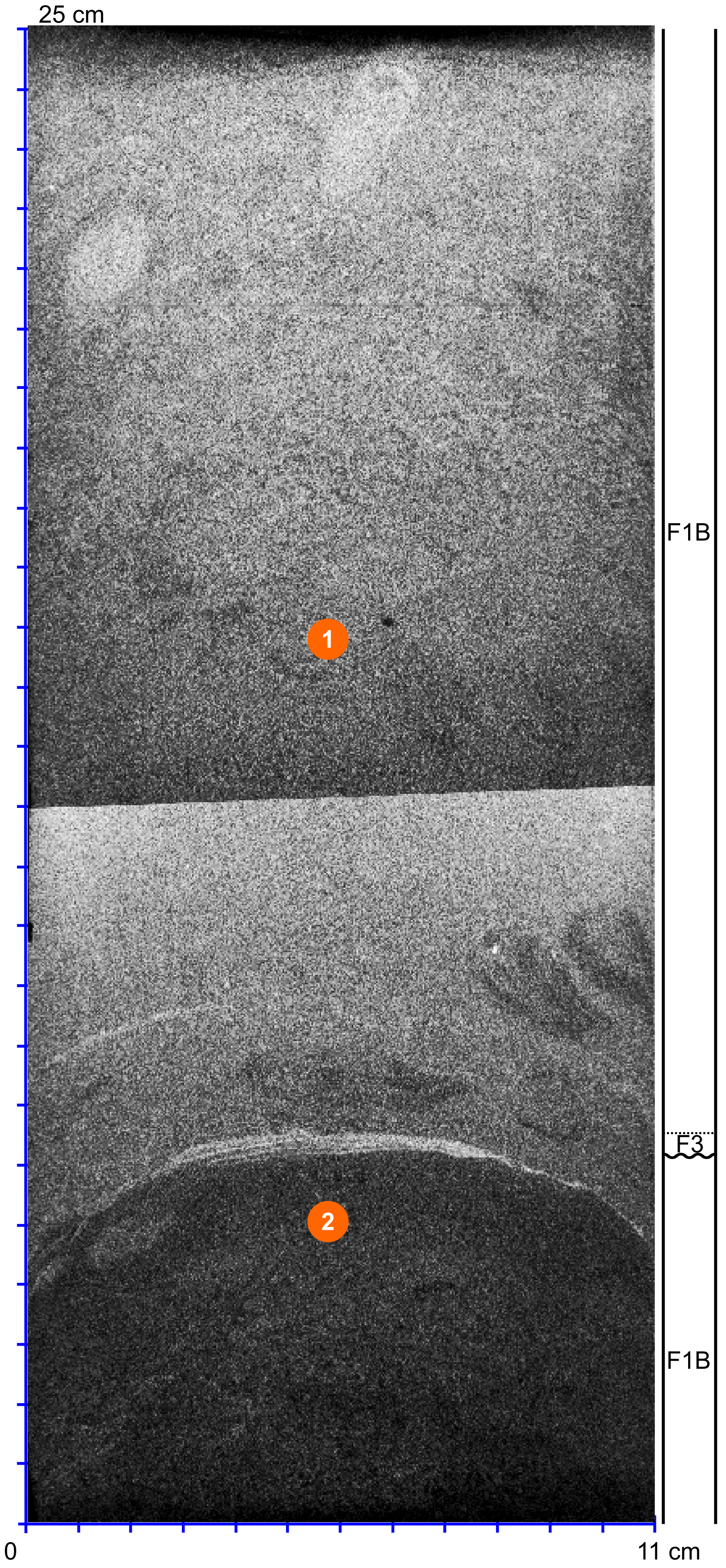

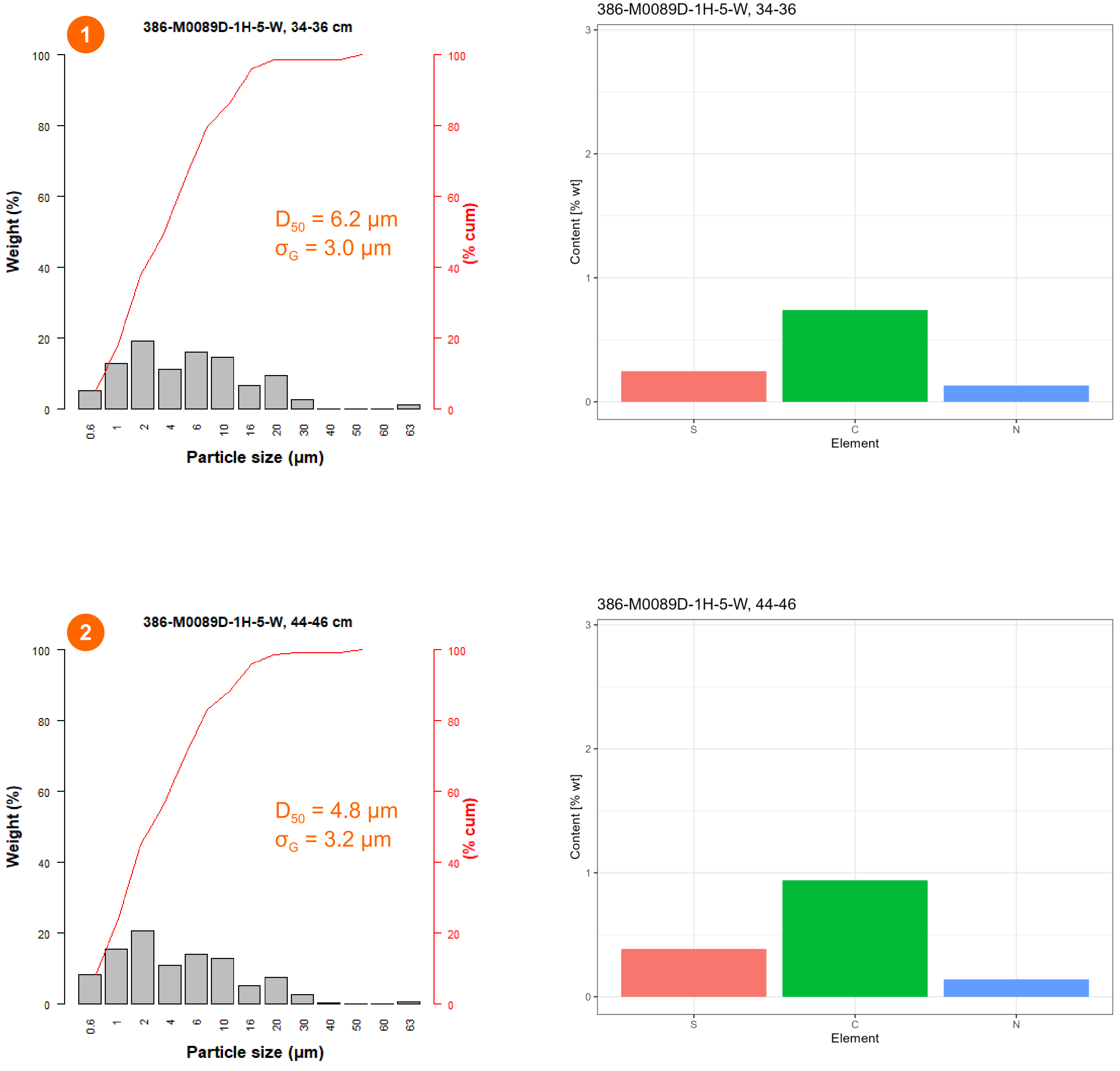

Supplementary Figure 15. CT image, facies and grain size distribution and sulphur, carbon and nitrogen content plots of the whole-round sample M0089D-1H-5WR36, 25–50 cm

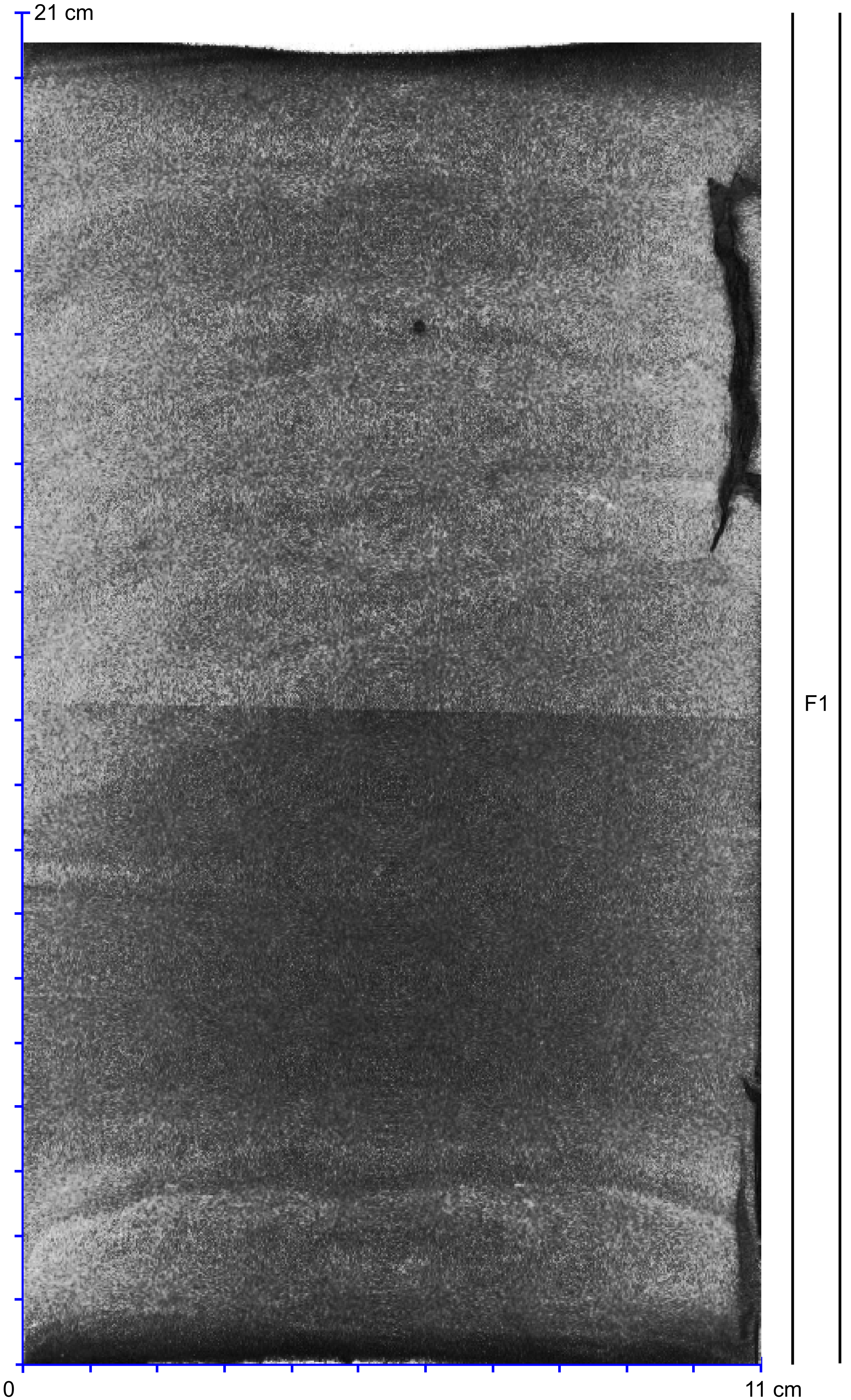

Supplementary Figure 16. CT image and facies of the whole-round sample M0081D-1H-5WR1, 80–100.5 cm

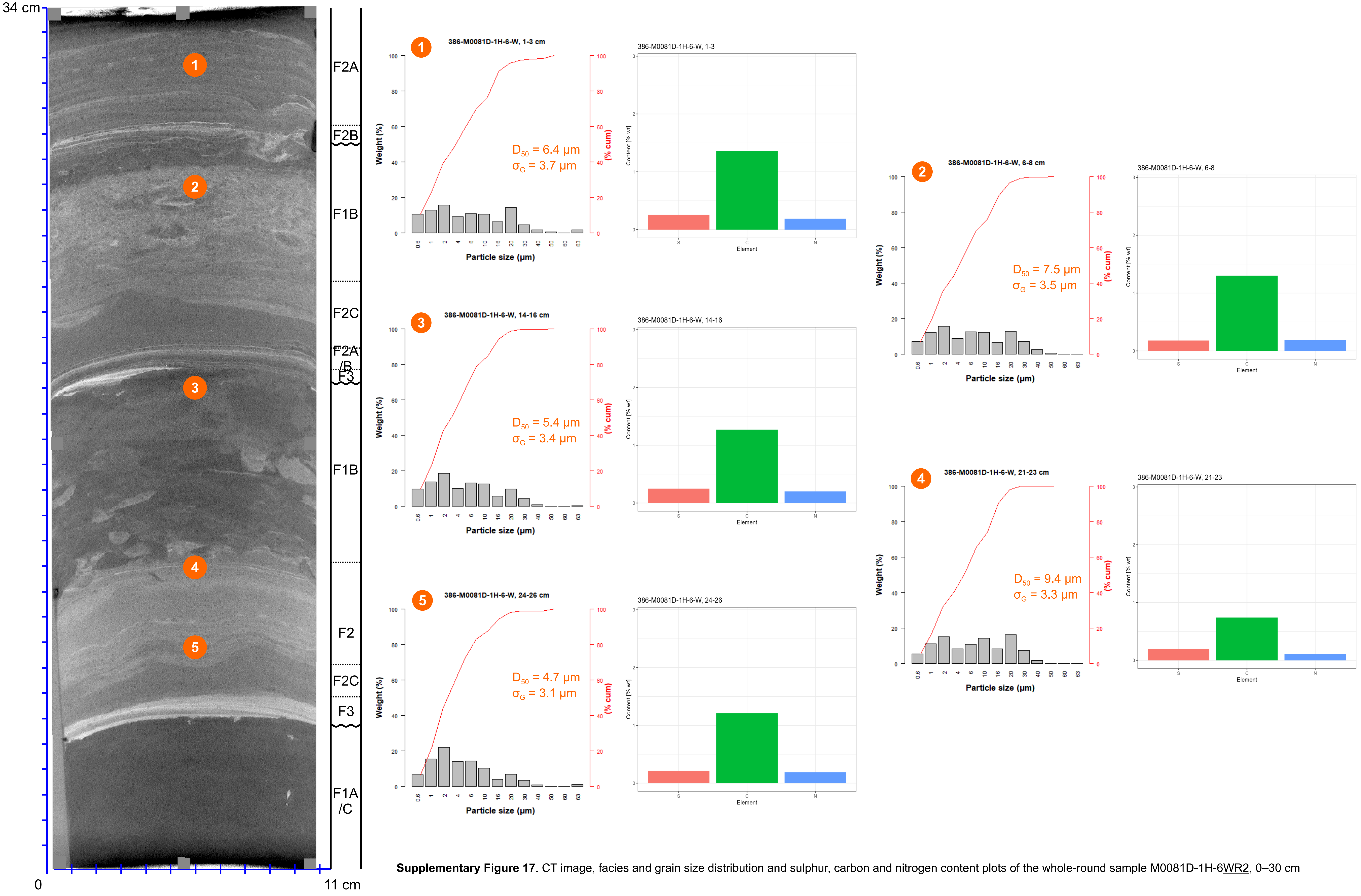

Supplementary Figure 17. CT image, facies and grain size distribution and sulphur, carbon and nitrogen content plots of the whole-round sample M0081D-1H-6WR2, 0–30 cm

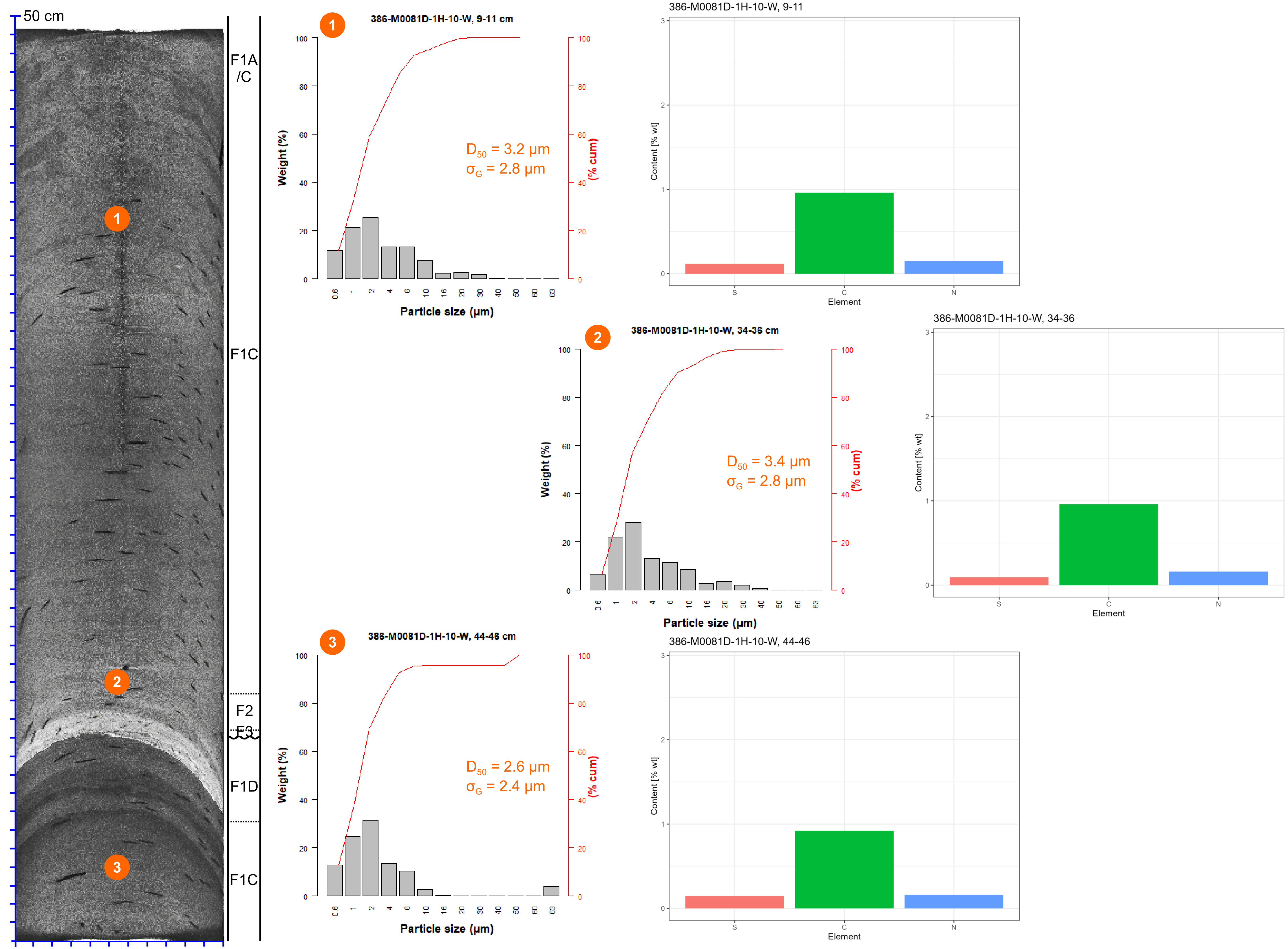

Supplementary Figure 18. CT image, facies and grain size distribution and sulphur, carbon and nitrogen content plots of the whole-round sample M0081D-1H-10WR3, 0–50 cm

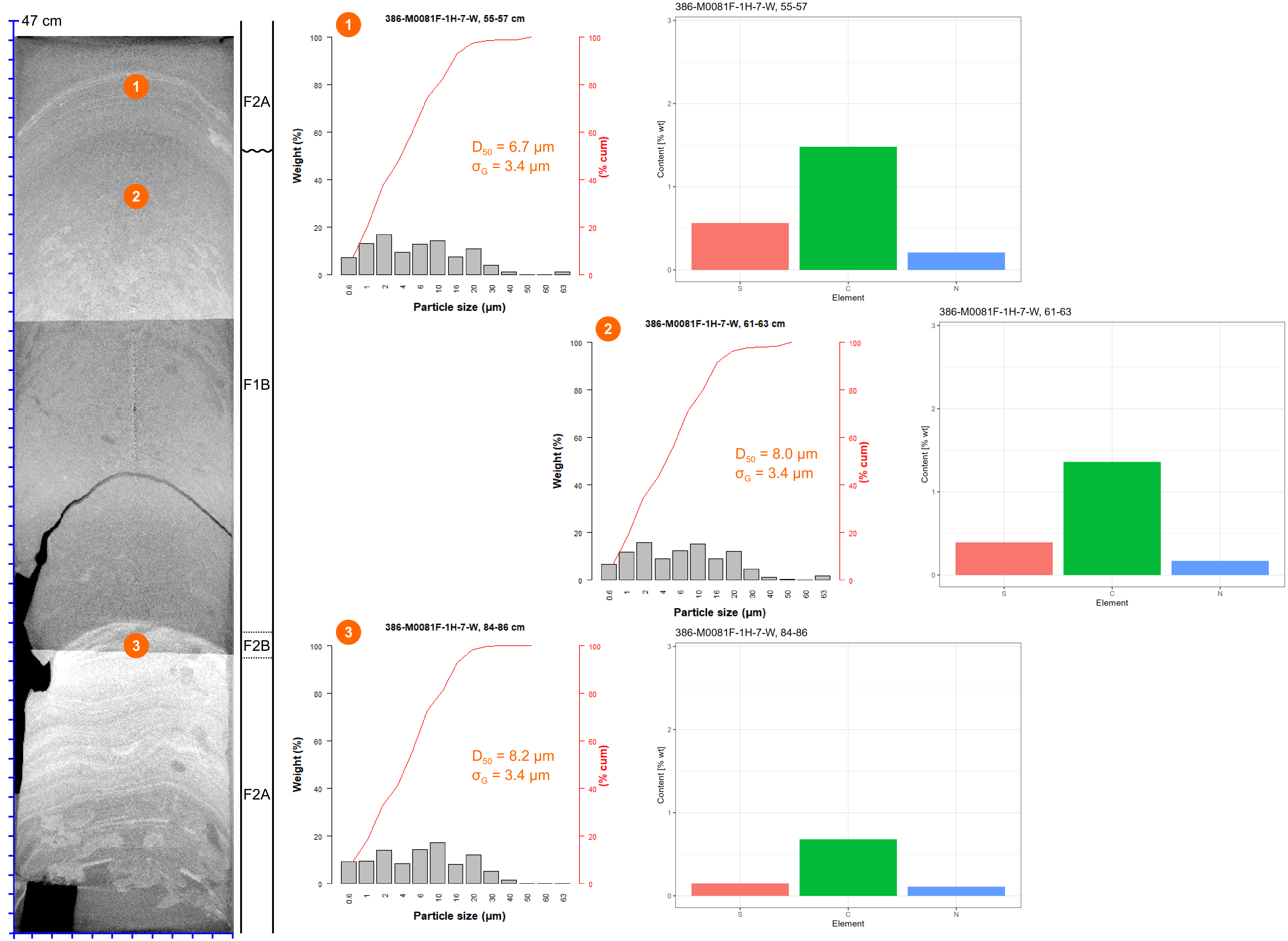

Supplementary Figure 19. CT image, facies and grain size distribution and sulphur, carbon and nitrogen content plots of the whole-round sample M0081F-1H-7WR4, 55–101.5 cm

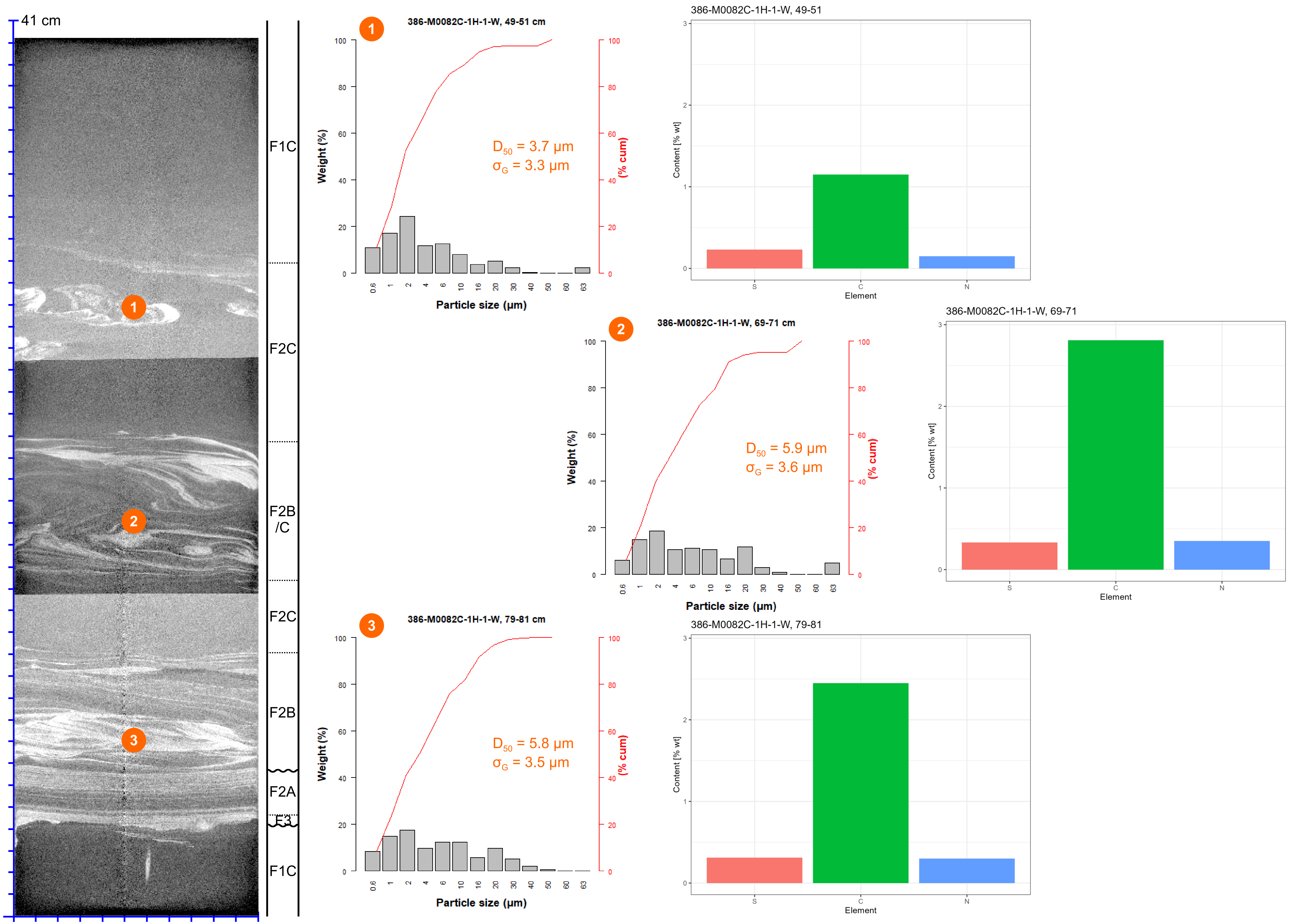

Supplementary Figure 20. CT image, facies and grain size distribution and sulphur, carbon and nitrogen content plots of the whole-round sample M0082C-1P-1WR6, 45–87 cm

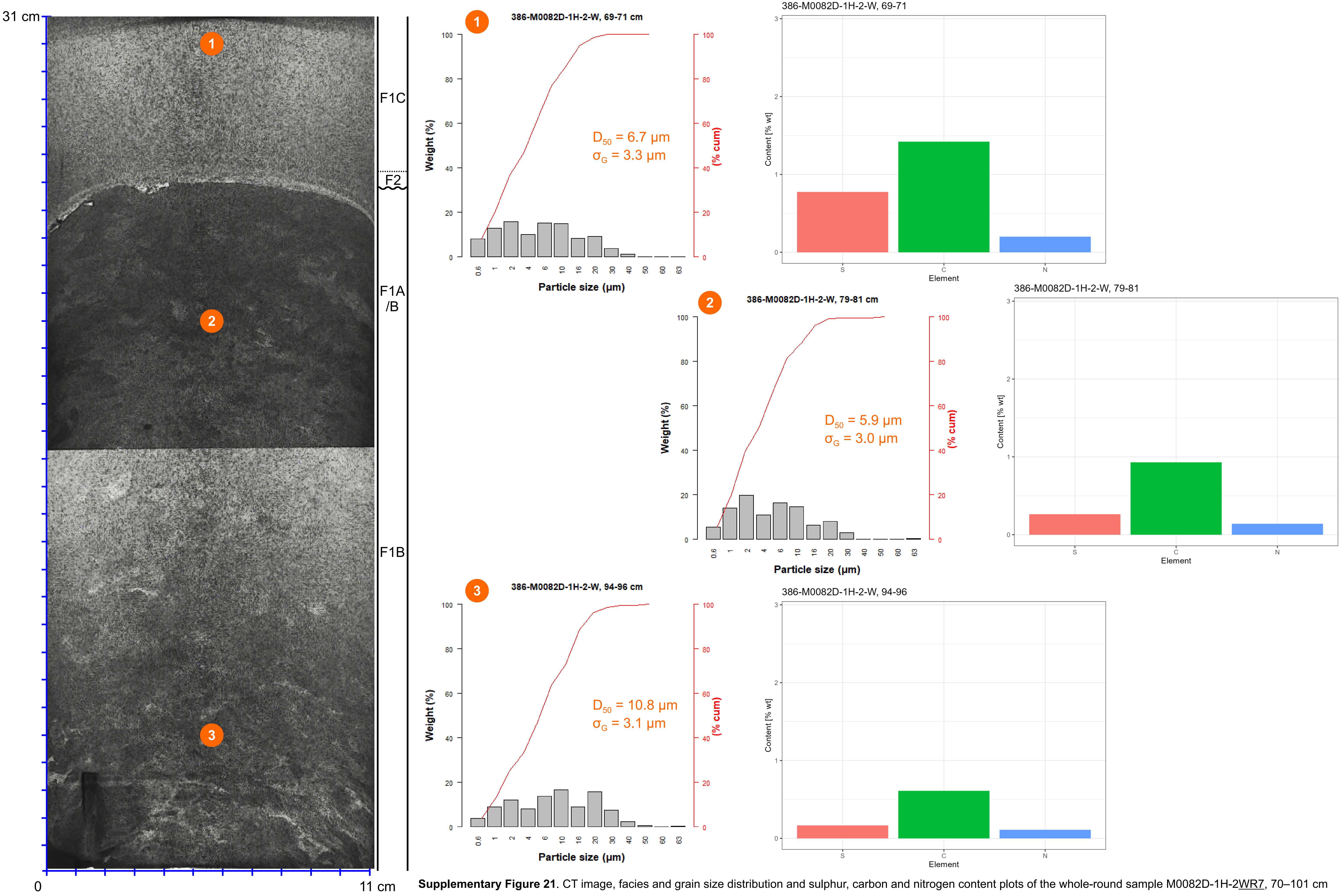

Supplementary Figure 21. CT image, facies and grain size distribution and sulphur, carbon and nitrogen content plots of the whole-round sample M0082D-1H-2WR7, 70–101 cm

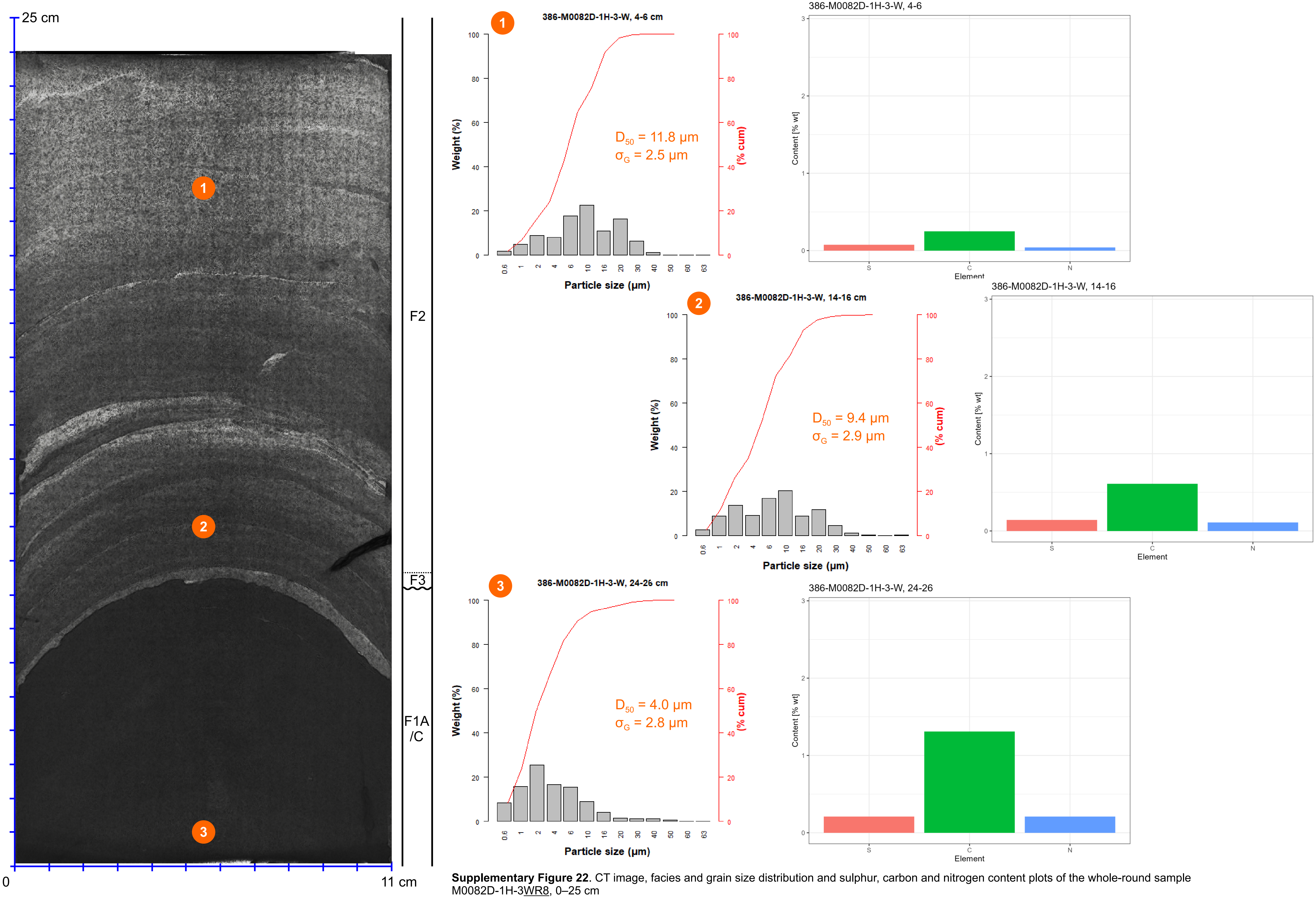

Supplementary Figure 22. CT image, facies and grain size distribution and sulphur, carbon and nitrogen content plots of the whole-round sample M0082D-1H-3WR8, 0–25 cm

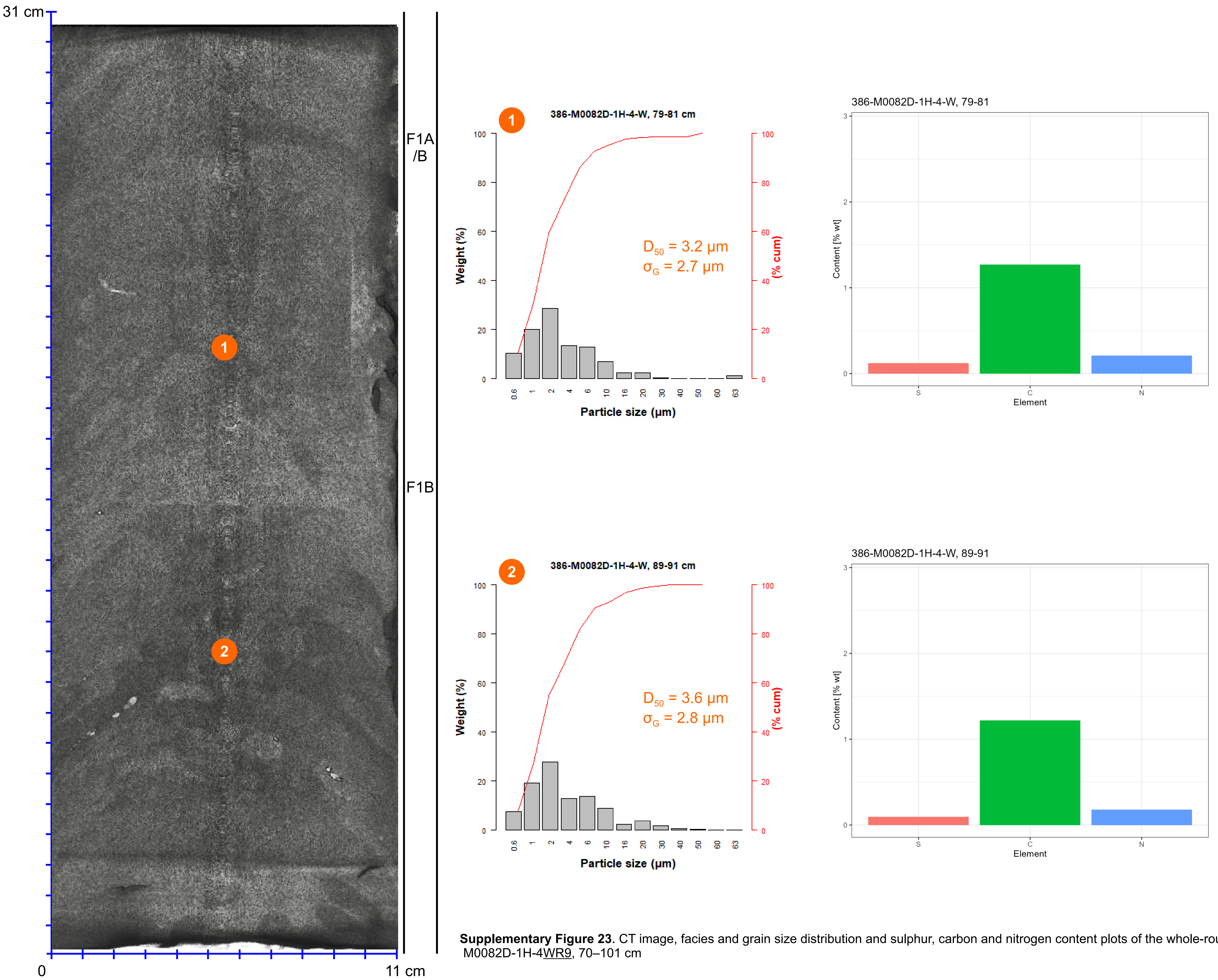

Supplementary Figure 23. CT image, facies and grain size distribution and sulphur, carbon and nitrogen content plots of the whole-round sample M0082D-1H-4WR9, 70–101 cm

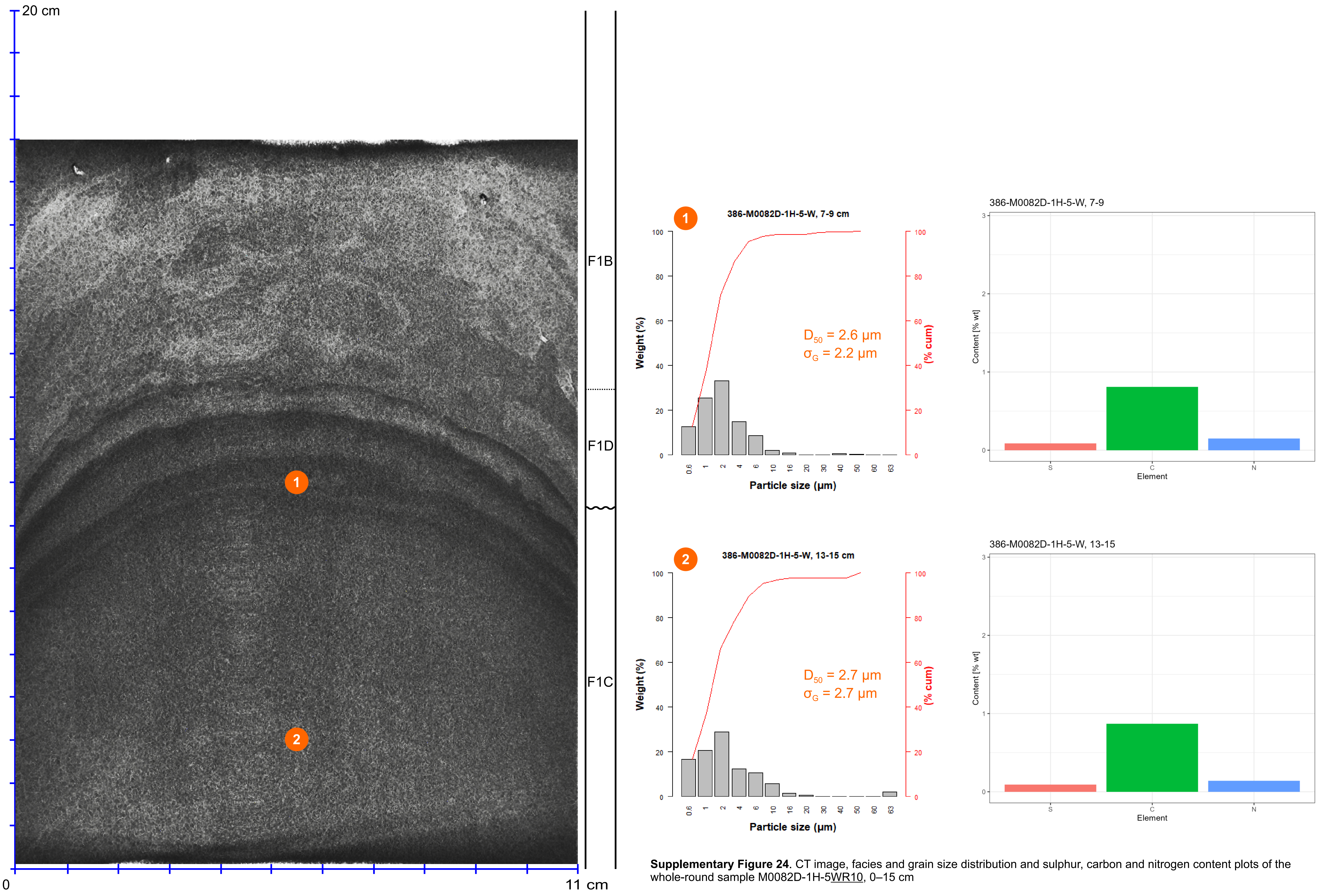

Supplementary Figure 24. CT image, facies and grain size distribution and sulphur, carbon and nitrogen content plots of the whole-round sample M0082D-1H-5WR10, 0–15 cm

Supplementary Note 4

Supplementary Table 3. Incipient trace fossil table

**Ichnogenus/ichnofamily**	**Description**	**Whole-Round (WR) core section**
Gyrolithes<br><br>Figs. 4A, B<br>Supplementary Figures 25A, B<br>Supplementary Movie 2	Helicoidal, corkscrew-like tube arranged in whorls around a sub-vertical to vertical axis. The whorls are not in contact. The tube fill is homogeneous. The tube has a circular to elliptical cross-section. Vertically, the longest preserved burrow extends for 29.1 mm and comprises 6 whorls. The diameter of complete whorls ranges from 1.7 to 4.1 mm (mean = 2.8 mm, n = 14). The tube diameter ranges from 0.5 to 1.1 mm (mean = 0.8 mm, n = 16). The burrows generally occur individually but, in a few cases (WR35), the corkscrew shows a first-order branching. The burrow has higher density than the mudstone possibly due to the enrichment in dense iron-bearing minerals such as pyrite.	7, 16, 17, 24, 27, 36
Nereites<br><br>Fig. 3E<br>Supplementary Figures 25B, C, D	Predominantly horizontal burrows, consisting of a core of backfilled dark (less dense) fine-grained material that is surrounded by a halo of coarser-grained sediment that is brighter (denser) than the backfilled core and the host sediment. The core is ~3 mm wide whereas the mantle is ~7 mm wide.	2, 4, 7, 8, 27, 35, 36
Phycosiphon<br><br>Fig. 3D<br>Supplementary Figures 25B, E	Small, U-shaped spreite burrow consisting of a dark, finer grained tube surrounded by pale coarser grained mantle. The U-shaped tube borders the spreite that is enriched in coarser grained material. The tubes typically appear dot- or hook-shaped, are 0.8–1.2 mm wide and enveloped by lighter material.	7, 8, 35, 36
Pilichnus<br><br>Figs. 4D, E, F<br>Supplementary Figures 25F, G<br>Supplementary Movie 4	Extensive burrow systems of 1–2 mm wide tubes, penetrating 5–10 cm deep with mostly 1st and 2nd order, irregular and variably oriented branching that form root-like, several cm-long extensions [5,6]. Locally, the deepest burrow extensions in the here documented burrows may show narrow helicoidal segments.<br><br>The branching tube system is closely associated or connected with a few-mm wide, straight to J-shaped or winding, 2–4 cm long, vertical to inclined shafts. The tubes show commonly irregular margin.	1, 2?, 3, 4, 6, 7, 9, 16, 17, 27?, 34, 35, 36
Rhizocorallium<br>Figs. 3B, C<br>Supplementary Fig. 25H<br>Supplementary Movie 1	U-shaped tube generating a protrusive spreite, oblique to the bedding. The trace is 15 mm wide and 30 mm long.	2

Continues...

...continued

Ichnogenus/ichnofamily	Description	Whole-Round (WR) core section
Rosselichnid ichnofamily<br><br>Figs. 3D–H<br>Supplementary Figures 25I–N	Horizontally to sub-vertically oriented burrows with a variably concentric or eccentric lamination enveloping a central tube[7].<br><br>Recurring burrow morphologies include:<br>- Dominantly horizontal shoe-shaped traces, which are 7–12 mm in diameter and display a central, finer-grained fill surrounded by irregular, concentric laminae. Occasionally, a package of laminae is developed at the base of the burrow appearing as a (subordinate) retrusive spreite. The central fill is best visible where the short vertical shaft turns horizontal. Some of the burrows show cross-section typical of *Artichnus*[8,9].<br>- Sub-vertical to inclined, variably laminated bow-shaped burrows, 7–10 mm in diameter. The burrows can occur solitarily bearing similarities with *Cylindrichnus* or form overlapping or closely spaced bundles, bearing locally transitional forms to *Phycodes*.<br>- Sub-vertical, spindle-shaped structure with a central tube surrounded by concentric laminae. The laminae tone (density) alternates between material similar to the matrix and darker, finer-grained and probably more organic-rich material. The internal diameter of the central tube ranges 1.0–2.5 mm; the tube wall is <1 mm thick. The lamina thickness ranges between 2.0 and 7.5 mm, and the diameter of the whole structure may reach 15 mm.<br>- Downwards branching, 5–9 mm wide, muddy burrows with a coarser grained (silt?) central tube that is surrounded by finer grained (clay?) laminated mud.	2, 3, 4, 7, 8, 9, 10?, 16, 17, 24, 26, 27, 34, 36<br><br><br>2, 4, 7, 8, 16, 26, 27, 34, 36<br><br><br>7, 27, 36<br><br>36<br><br><br>4
Trichichnus<br><br>Fig. 4C, Supplementary Figure 25O	Vertical to oblique, straight or slightly winding, rarely branched cylindrical burrow, up to 1 mm in diameter. The contrast of the burrow is enhanced by its fill having a high iron sulphide content (greigite or pyrite)[10].	3, 4?, 9, 24, 34, 35, 36
Zoophycos-like<br><br>Fig. 4G and H<br>Supplementary Movie 3	Lobate burrows up to 5 cm high, showing a crescentic or massive-appearing fill structure. The lobes are narrow reaching a width of 13 mm, while their thickness is 1–3 mm. The lobes can be attached to a vertical helicoidally twisted shaft, 3 cm long and 1 mm in diameter in the top. Burrowing depth ranges from 3 to 7 cm.	2, 8, 16?
**Other:**<br>morphology/ethology		
Vertical shafts<br>Supplementary Figs. 25P, Q	Large, 4–17 cm-deep penetrating, vertical, straight to variably bending shafts, ca. 2–4 mm in diameter. The burrows can show a single downward-oriented y-shaped branch.	8, 9
fugichnia<br>*Fig. 3A*	Irregular, horizontal to vertical, funnel-shaped trace with deformed and diffuse burrow margins	27

Supplementary Figure 25. Incipient trace fossil images

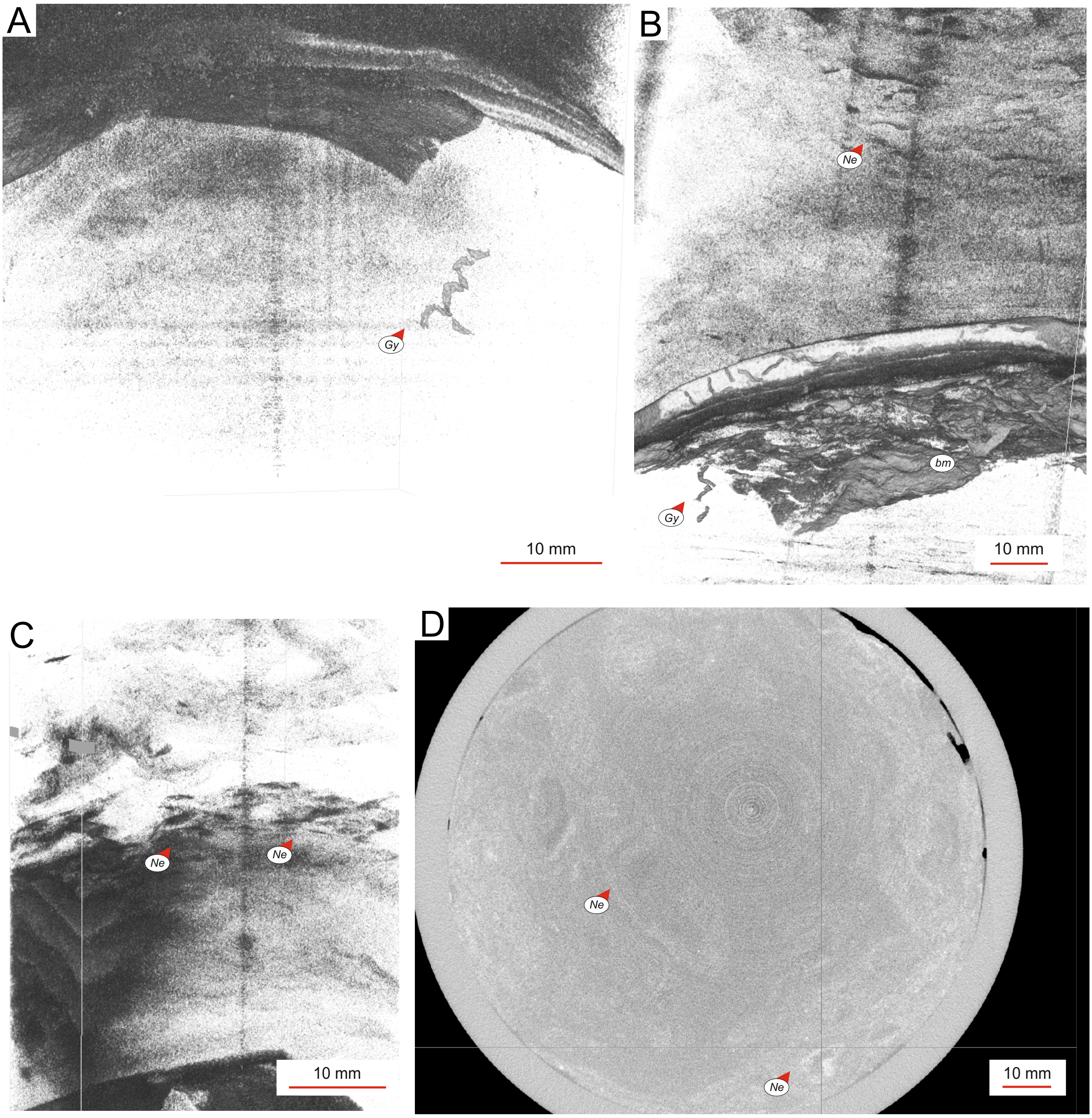

A) and B) *Gyrolithes* (*Gy*), *Nereites* (*Ne*) and horizontal burrow mottling (bm). A: M0089D-1H-5WR36, 25–50 cm and B: M0084F-1H-21WR24, 35–45 cm. C) and D) Vertical and horizontal (top-down) view to *Nereites*. M0081D-1H-6WR2, 15–20 cm. Note that in the 3D view, the provided scale bar gives an approximate scale for features in the foreground of the figure.

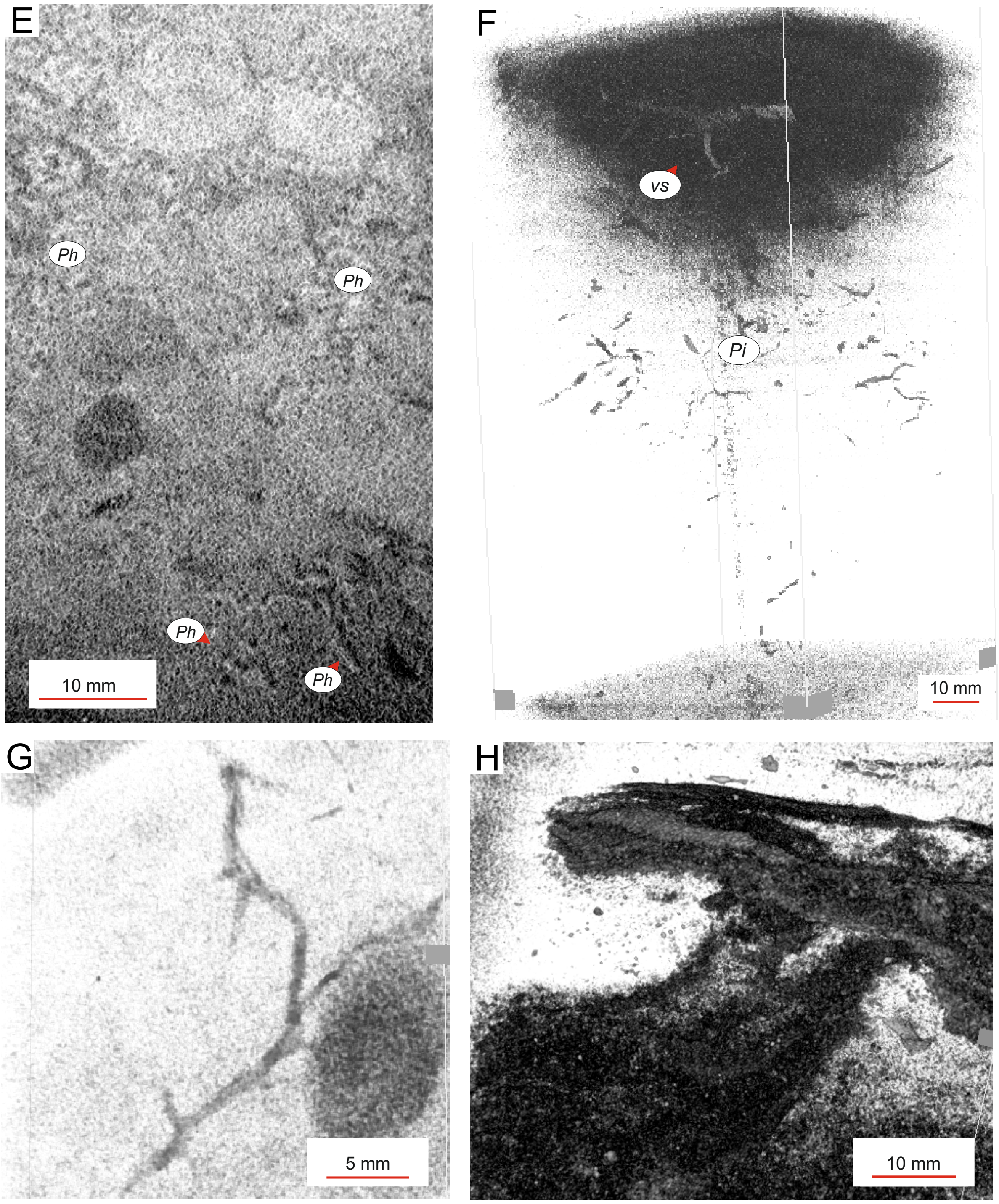

E) *Phycosiphon* fabric (Ph) cross-cut by potential Rosselichnid burrows (see article figure 3D). F) A high-contrast image of *Pilichnus* (Pi). Note the associated vertical element (vs) at the top of the burrow system. M0089D-1H-5WR35, 0– ~15 cm. G) A top-down view to *Pilichnus*. M0089D-1H-5WR36, 25–30 cm. H) *Rhizocorallium*. Core section M0081D-1H-6WR2, 4.5–6.5 cm. Note that in the 3D view, the provided scale bar gives an approximate scale for features in the foreground of the figure.

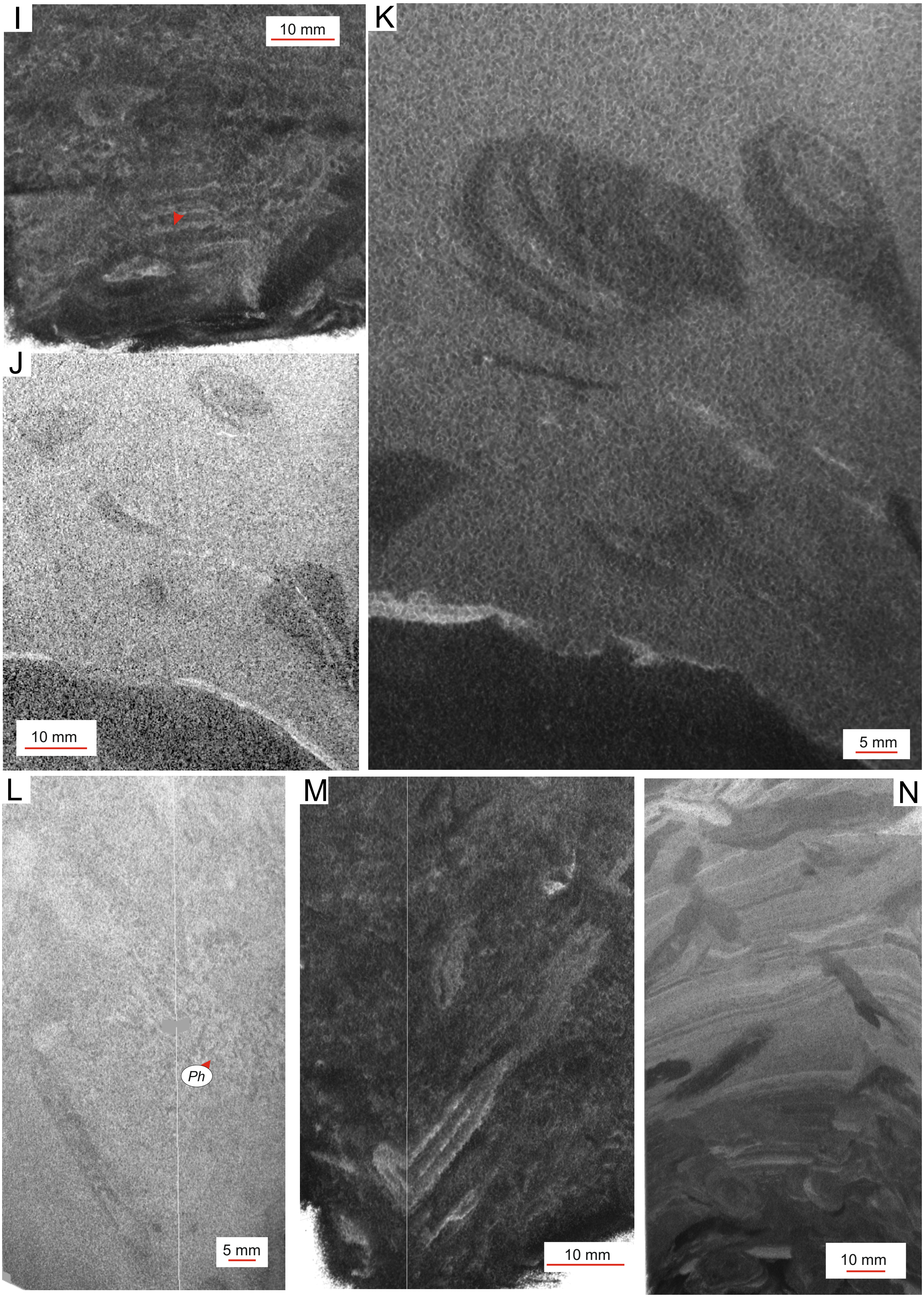

Burrows assigned to the Rosselichnid ichnofamily. I) *Artichnus*-like (red arrow) burrow cross-cutting *Nereites* fabric. M0082D-1H-2WR7, 95–101 cm. J and K) A spindle-shaped and variably laminated burrows. Note that the core margin is bent downwards due to the coring process in both images. M0089D-1H-5WR36, 38–45cm. L) and M) Sub-vertical burrows cross-cutting *Phycosiphon* and *Nereites*, respectively. The example in Figure M shows overlapping/closely spaced bundles being a transitional form to *Phycodes*. L: M0089D-1H-5WR36, 25–35 cm. M: M0085D-1H-3WR27, ~93– ~100 cm. N) Downwards branching muddy burrows with a coarser grained (silt?) central tube. M0081F-1H-7WR4, 87–101 cm. Note that in the 3D view, the provided scale bar gives an approximate scale for features in the foreground of the figure.

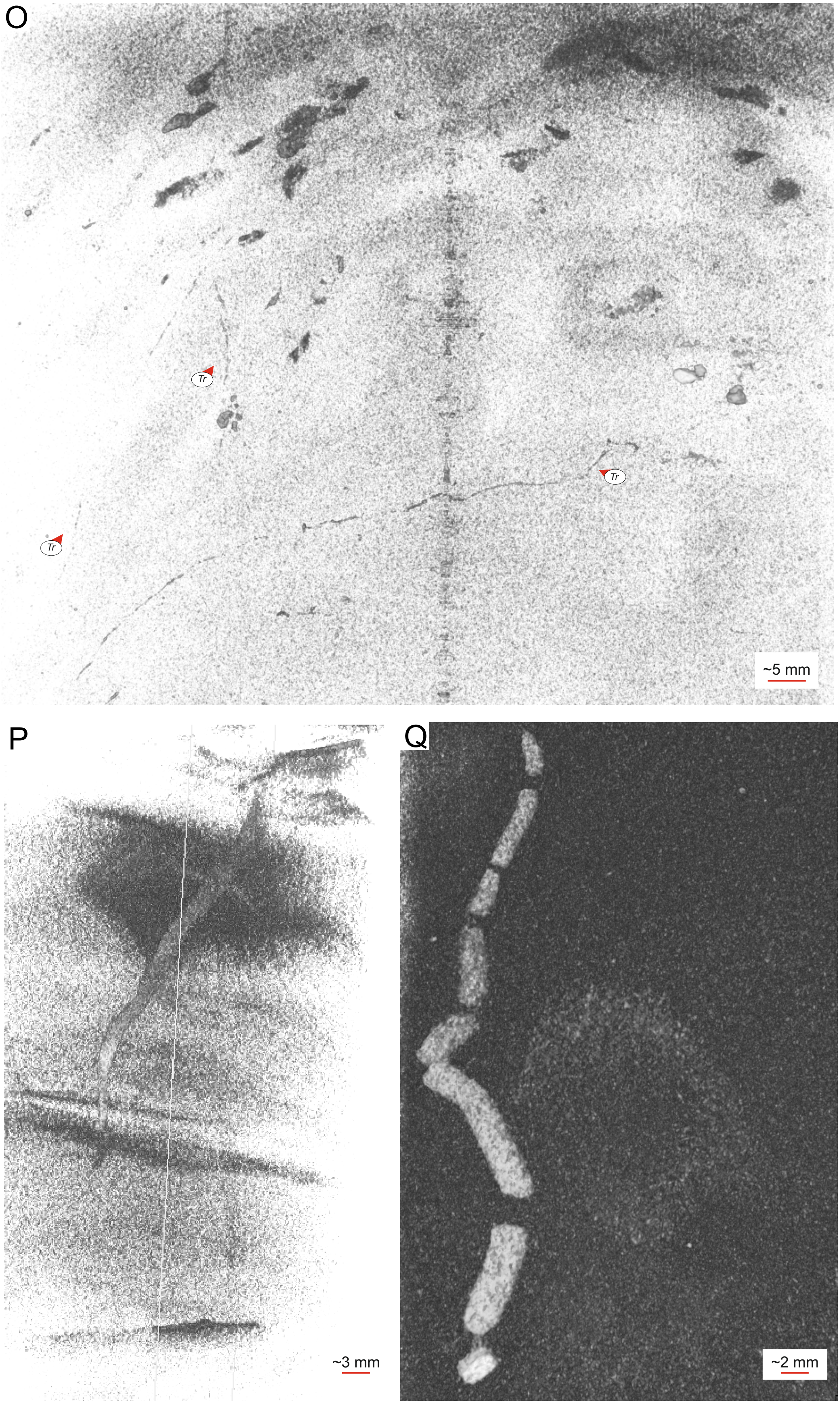

O) Variably oriented examples of *Tricichnus* (*Tr*). The burrow diameter is <1 mm. M0081D-1H-10WR3, 0– ~10 cm. P and Q) Larger-scale vertical shafts. M0082D-1H-3WR8, 0–5 cm and M0082D-1H-4WR9, 82–88 cm. Note that in the 3D view, the provided scale bar gives an approximate scale for features in the foreground of the figure.

Supplementary Note 5

Supplementary Figure 26. Plots of measured bioturbation properties in the trench basins

Vertical bars indicate interquartile ranges. Median value points are connected by a blue line. The numbers are p-values of Wilcoxon rank sum tests with Bonferroni correction for multiple comparisons. n = number of observations. Source data are available as a Source Data file.

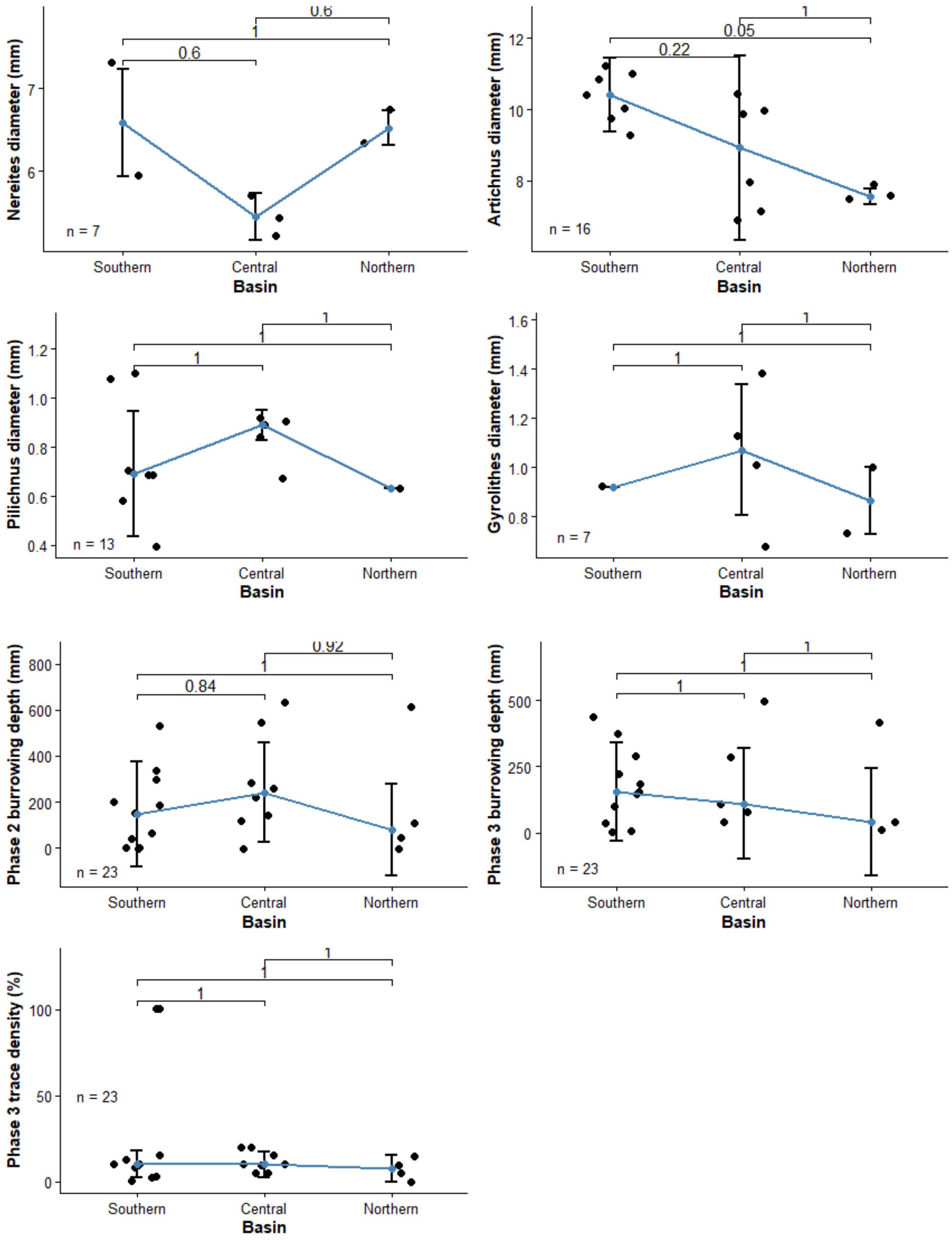

Supplementary References

1) Strasser, M., Ikehara, K., Pizer, C., Itaki, T., Satoguchi, Y., Kioka, A., ... & Zellers, S. D., 2024. Japan Trench event stratigraphy: First results from IODP giant piston coring in a deep-sea trench to advance subduction zone paleoseismology. Marine Geology, 477, 107387.
2) Talling, P.J., Masson, D.G., Sumner, E.J., Malgesini, G., 2012. Subaqueous sediment density flows: depositional processes and deposit types. Sedimentology 59, 1937–2003.
3) Stow, D.A.V., Shanmugam, G., 1980. Sequences of fine grained turbidites – comparison of recent deep-sea and ancient flysch sequences. Sedimentary Geology 25, 23–42.
4) Stow, D.A.V., Piper, D.J.W., 1984. Deep-water fine-grained sediments: facies models. In: Stow, D.A.V., Piper, D.J.W. (Eds.), Fine Grained Sediments, Deep-water Processes and Facies. Geological Society, London, Special Publication 15, 611–646.
5) Uchman, A. 1999. Ichnology of the Rhenodanubian Flysch (Lower Cretaceous-Eocene) in Austria and Germany. Beringeria 25, 65–171.
6) Mikuláš, R., Lehotský, T. & Bábek, O. 2004. Trace fossils of the Moravice Formation from the southern Nízký Jeseník Mts. (Lower Carboniferous, Culm facies; Moravia, Czech Republic). Bulletin of Geosciences 79, 81–98.
7) Knaust, D., 2021. Rosselichnidae ifam. nov.: burrows with concentric, spiral or eccentric lamination. Papers in Palaeontology 7, 1847–1875
8) Zhang, G., Uchman, A., Chodyń, R., Bromley, R.G., 2008. Trace fossil *Artichnus pholeoides* igen. nov. isp.nov. in Eocene turbidites, Polish Carpathians: possible ascription to holothurians. Acta Geologica Polonica 58, 75–86.
9) Ayranci K., Dashtgard, S.E., 2013. Infaunal holothurian distributions and their traces in the Fraser River delta front and prodelta, British Columbia, Canada. Palaeogeography, Palaeoclimatology, Palaeoecology 392, 232–246.
10) McBride, E.F, Picard, M.D., 1991. Facies implications of *Trichichnus* and *Chondrites* in turbidites and hemipelagites, Marnosoarenacea Formation (Miocene), Northern Apennines, Italy. Palaios 6. 281–290.
